# Perioperative esketamine for prevention of postoperative sleep disturbance after anesthesia: a systematic review and meta-analysis of randomized controlled trials

**DOI:** 10.3389/fphar.2026.1852647

**Published:** 2026-06-03

**Authors:** Jinfang Zeng, Jinzhi Li, Yonglian Zhou, Shu Yang, Jinjin Jian, Minmin Zhu

**Affiliations:** 1 Department of Anesthesiology and Pain, Jiangnan University Medical Center (Wuxi No. 2 People’s Hospital, Affiliated Wuxi Clinical College of Nantong University), Wuxi, China; 2 Department of Anesthesiology, Quanzhou Hospital of Traditional Chinese Medicine, Quanzhou, China; 3 Wuxi School of Medicine, Jiangnan University, Wuxi, China; 4 Department of Anesthesiology, Affiliated Hospital of Jiangnan University, Wuxi, China

**Keywords:** esketamine, meta-analysis, postoperative sleep disturbance, randomized controlled trial, systematic review

## Abstract

**Background:**

Postoperative sleep disturbance (PSD) is a common complication after general anesthesia and is associated with delayed recovery and other adverse postoperative outcomes. Perioperative esketamine may improve postoperative sleep, but current evidence remains inconclusive. This systematic review and meta-analysis assessed the efficacy and safety of perioperative esketamine for preventing PSD in adults undergoing surgery under general anesthesia.

**Methods:**

PubMed, Embase, the Cochrane Library, and CNKI were searched from inception to 10 March 2026, for randomized controlled trials comparing perioperative esketamine with placebo or non-esketamine controls. The primary outcomes were the incidence of Postoperative sleep disturbance on postoperative day 1, 2, 3, and 7. Secondary outcomes included common perioperative adverse events. Risk ratios (RRs) with 95% confidence intervals were pooled using a random-effects model. Risk of bias, certainty of evidence, and robustness of key findings were assessed using the Cochrane Risk of Bias tool, GRADE, trial sequential analysis, and leave-one-out sensitivity analyses.

**Results:**

Twenty-two randomized controlled trials were included. Esketamine significantly reduced PSD incidence on POD1 (RR = 0.58, 95% CI: 0.51–0.66; I^2^ = 0%), POD2 (RR = 0.40, 95% CI: 0.28–0.58; I^2^ = 0%), and POD3 (RR = 0.55, 95% CI: 0.45–0.68; I^2^ = 9%), but not on POD7 (RR = 0.77, 95% CI: 0.55–1.08). Although statistical heterogeneity was low, clinical heterogeneity should be considered because of differences in surgical populations, patient characteristics, esketamine regimens, and sleep assessment methods. Subgroup analyses generally supported the early postoperative benefit, whereas POD3 findings stratified by preoperative sleep status should be interpreted cautiously. No statistically significant differences were observed in the analyzed adverse events, but safety evidence remains limited. Sensitivity analyses and trial sequential analysis supported the robustness of POD1 and POD3 findings. The certainty of evidence for early PSD outcomes was moderate.

**Conclusion:**

Perioperative esketamine may reduce early PSD incidence, particularly from POD1 to POD3. However, evidence mainly reflects subjective binary PSD outcomes, while effects on objective sleep parameters and longer-term sleep recovery remain uncertain. Further high-quality, multicenter trials using standardized and objective sleep assessments are needed.

**Clinical Trial Registration:**

PROSPERO (CRD420261364623).

## 1 Introduction

Postoperative sleep disturbance (PSD) is a frequent postoperative complication, with an incidence reported to range from 15% to 72% depending on the study and surgical population (Gao et al., 2022). Its clinical manifestations include poor sleep quality, reduced sleep duration, circadian rhythm disruption, and changes in normal sleep structure (Nelson et al., 2025; Yuan et al., 2024). Increasing evidence indicates that PSD is associated with a range of unfavorable postoperative outcomes, such as heightened pain perception, delirium, cognitive decline, impaired immune response, slower functional recovery, and longer hospitalization (Niklasson et al., 2025; Guo et al., 2024). Recently, perioperative sleep disorders have become a research focus at home and abroad. Various pharmacological approaches, such as melatonin, dexmedetomidine and zolpidem have been explored for this (T et al., 2025; Nithagon et al., 2025). Despite this, their clinical utility is limited by variable efficacy, negative impact on hemodynamic stability, potential disruption of normal sleep architecture and relatively modest effect on pathophysiological processes leading to PSD.

Sleep regulation is closely related to the balance between excitability and inhibitory nerve transmission in the central nervous system. N-methyl-D-aspartic acid (NMDA) receptor is an ionized glutamate receptor involved in excitatory synaptic transmission and sleep-wake regulation; recent experimental evidence shows that NMDA receptors in the hypothalamic lateral anterior view are crucial to maintaining NREM and REM sleep (Miracca et al., 2022). During the perioperative period, surgical pressure, postoperative pain, inflammation and exposure to opioids may lead to sleep fragmentation, reduced sleep continuity and impaired sleep structure (Huang et al., 2025). Therefore, the regulation of NMDA receptor-mediated signaling provides a biologically reasonable mechanism. Esketamin during surgery may affect postoperative sleep, especially in the early postoperative period (Liu et al., 2025).

Derived as the S-enantiomer of racemic ketamine (RK), esketamine acts as a noncompetitive antagonist at the N-methyl-D-aspartate receptor and is characterized by enhanced analgesic efficacy and more favorable tolerability at subanesthetic doses (Mio et al., 2024; Jelen et al., 2021; Liu et al., 2023). Esketamine has garnered considerable interest as an anesthetic adjunct in perioperative settings given its ability to mitigate postoperative pain and opioid requirements, and may also benefit affective symptoms intimately associated with PSD (Duncan and Zarate, 2013; Qiu et al., 2022; Chen et al., 2024). Mechanistically, ketamine-derived compounds seem to act on sleep in part by modulating sleep architecture (in particular, slow-wave activity) as well as pathways implicated in synaptic plasticity and brain-derived neurotrophic factor signalling (Duncan and Zarate, 2013; Qiu et al., 2022; Song and Zhu, 2021). Clinical data have also implicated a potential role for perioperative esketamine in enhancing postoperative sleep. PSD is reduced after gynecological laparoscopy and surgical abortion in randomized trials (Song et al., 2025). However, not all studies have observed a clear advantage: A recent trial in patients who underwent modified radical mastectomy failed to show a significant benefit on postoperative sleep quality (Chen Y. et al., 2025). Collectively, these findings suggest that esketamine might be a promising intervention for PSD; however, the variability in surgical populations, dosing regimens, baseline sleep status, analgesic strategies, and sleep assessment methods highlights the need for an integrated synthesis of randomized clinical evidence.

Due to the conflicting evidence regarding perioperative esketamine, we performed this systematic review and meta-analysis to assess the efficacy and safety of esketamine in preventing PSD after general anesthesia. To the best of our knowledge, we specifically investigated sleep outcomes at various postoperative time points and included the adverse event rate.

## 2 Methods

In accordance with standard guidelines, ethical approval was not required for this systematic review.

### 2.1 Study registration

Using a systematic review and meta-analysis, this study aimed to evaluate whether perioperative esketamine affects PSD in patients receiving general anesthesia. This study adhered to the Preferred Reporting Items for Systematic Reviews and Meta-Analyses (PRISMA) statement. A protocol for this systematic review was prospectively registered with the International Prospective Register of Systematic Reviews (PROSPERO; registration number CRD420261364623).

### 2.2 Search strategy and eligibility criteria

PubMed, Embase, Cochrane Library, and CNKI were systematically searched for relevant studies from inception to 10 March 2026. The search was conducted combining subject headings and free-text terms relevant to esketamine, postoperative sleep (e.g., “esketamine,” “S-ketamine,” “(S)-ketamine,” “postoperative sleep disturbance,” “sleep quality,” “insomnia,” and “perioperative care” and general anesthesia). Boolean operators were then employed to combine these terms as per the individual database requirements. Search strategies in full are reported in Supplementary Table S1. To capture all potentially relevant studies, the reference lists of included publications and relevant reviews were manually reviewed.

### 2.3 Research selection

Following the removal of duplicate records, two reviewers independently screened the titles and abstracts of the retrieved studies. Potentially relevant articles were then assessed in full text against the predefined eligibility criteria. Disagreements arising during study selection were addressed through discussion and resolved by consensus.

Using a standardized extraction form, data were obtained from all eligible studies. The extracted items covered basic study information, including first author, year of publication, and country; participant and surgical characteristics, such as sample size, age, sex distribution, and surgical type; and details of anesthesia and intervention protocols, including the timing, route, and dose of perioperative esketamine as well as the control regimen. Outcome data were also recorded. Particular attention was paid to the incidence of PSD at different postoperative time points. Because sleep disturbance was assessed with different instruments across trials, we prespecified a hierarchical extraction strategy for the dichotomous PSD outcome. When a trial explicitly reported the number of patients with PSD at a given postoperative time point, these event data were extracted directly. When the trial defined PSD using a validated cutoff, such as AIS ≥6, AIS >6, NRS-sleep ≥6, or PSQI ≥5, the event numbers based on the authors’ original definition were used. Continuous sleep-scale scores, including AIS, PSQI, RCSQ, NRS-sleep scores, or actigraphy-derived parameters, were not converted into dichotomous PSD events unless the original trial explicitly reported the corresponding number of patients meeting the prespecified PSD definition. Trials that reported only continuous sleep outcomes without a binary PSD incidence were summarized descriptively and were not included in the risk-ratio meta-analysis for PSD incidence. When reported, additional information on sleep assessment methods, preoperative sleep status, and adverse events, including postoperative nausea and vomiting, bradycardia, dizziness, headache, nightmare, and hypertension, was also extracted.

### 2.4 Inclusion criteria

Eligibility for studies required meeting all of the following criteria: (1) they must be randomized controlled trials; (2) adult patients undergoing surgery; (3) surgery performed under general anesthesia; (4) perioperative use of esketamine, including S-ketamine or (S)-ketamine, as the intervention; and (5) reporting at least one postoperative sleep-related outcome, such as the incidence of PSD or postoperative sleep quality measured with a validated assessment tool. No limitations were imposed on the type of surgery or on the timing, route, or dosage of perioperative esketamine administration.

### 2.5 Exclusion criteria

Studies were excluded if they met any of the following criteria: (1) non-randomized study design; (2) inclusion of non-surgical patients; (3) esketamine not administered during the perioperative period; (4) procedures not performed under general anesthesia; (5) esketamine used primarily for the management of depression or other psychiatric conditions; or (6) failure to report any postoperative sleep-related outcome. For studies based on overlapping populations, only the report providing the most complete data was retained for analysis.

### 2.6 Information extraction and evaluation of bias risk

Employing a standardized form, two investigators (Z.J.F. and J.J.J.) independently gathered data from the eligible studies and assessed each trial’s quality. The extracted data included study characteristics, patient demographics, surgical and anesthetic information, details of the intervention (e.g., duration), control regimens, and postoperative sleep-related outcomes, as well as adverse events when reported.

To assess methodological quality, the Cochrane Collaboration Risk of Bias tool (ROB 1) was applied across various domains, including random sequence generation, allocation concealment, participant and personnel blinding, outcome assessment blinding, incomplete outcome data, selective reporting, and other possible biases. Ratings for each domain were assigned as low, unclear, or high risk of bias, and conflicts were addressed through discussion until consensus was obtained.

### 2.7 Quality analysis of evidence

Evidence certainty for the principal outcomes was evaluated using the GRADE framework. Assessment of each outcome considered the domains of risk of bias, inconsistency, indirectness, imprecision, and, when relevant, potential publication bias. Evidence was assessed and classified into four levels: high, moderate, low, or very low. Summary of Findings tables were produced to summarize evidence certainty for PSDs at various time points and associated adverse events.

### 2.8 Trial sequential analysis

Using Trial Sequential Analysis software (version 0.9.5.10 beta), TSA was performed to examine the incidence of PSD on postoperative days 1 and 3. TSA was applied to evaluate whether the cumulative evidence was sufficiently robust and whether further trials were still needed, while reducing the risk of random errors caused by sparse data and repeated significance testing. The required information size was calculated with adjustment for between-study heterogeneity. A two-sided type I error of 5% and a type II error of 20% were predefined. The cumulative Z-curve was compared against sequential monitoring boundaries, and evidence was considered conclusive if it crossed the boundary before meeting the required information size. Otherwise, further studies were considered necessary to draw robust conclusions.

### 2.9 Outcome measures

Data analyses were carried out with Review Manager (RevMan, version 5.4) or Stata (version 17.0). Dichotomous outcomes, including PSD incidence and perioperative adverse events, were analyzed using pooled risk ratios (RRs) with 95% confidence intervals (CIs). For PSD incidence, the binary event data were extracted according to the definitions and cutoff values prespecified in the original trials. We did not standardize or transform different continuous sleep metrics into a single dichotomous outcome. Therefore, studies reporting only continuous sleep-scale scores or actigraphy-derived sleep parameters without explicit PSD event numbers were not included in the RR meta-analysis for PSD incidence. Objective or semi-objective sleep outcomes, such as actigraphy-derived total sleep time, sleep efficiency, wake after sleep onset, and EEG-derived sleep features, were summarized descriptively when reported, because the number of studies and reporting formats were insufficient for quantitative pooling. The sleep assessment instruments, cutoff values, and operational definitions used in each included trial are summarized in Supplementary Table S2. Due to the varying postoperative time points of sleep disturbance reported (POD1, POD2, POD3 and POD7) between studies separate meta‐analyses were performed for each postoperative day. The I^2^ statistic was used to evaluate heterogeneity, and a two-sided P value below 0.05 was regarded as statistically significant. All analyses were prespecified and examined according to a random-effects framework. Prespecified subgroup analyses were performed for the primary sleep outcomes on POD1 and POD3 to understand possible sources of heterogeneity.

These analyses were stratified according to whether a loading or bolus dose was administered, the presence or absence of preoperative sleep disturbance, and the timing of esketamine administration (intraoperative only vs. postoperative-only or intraoperative plus postoperative). In addition, for POD1, a further subgroup analysis was performed according to maintenance infusion rate (<0.2 mg/kg/h, 0.2–0.3 mg/kg/h, and >0.3 mg/kg/h). Within subgroups, RRs were pooled, and 95% CIs were calculated. Heterogeneity was examined using the I^2^ statistic and Cochran’s Q test, and differences between subgroups were assessed using the test for subgroup differences. The stability of the pooled estimates for POD1 and POD3 was further explored through leave-one-out sensitivity analyses. When outcomes had enough studies, publication bias and small-study effects were investigated using funnel plots along with Begg’s and Egger’s tests, and trim-and-fill analysis was utilized when warranted. For trials with missing or unclear outcome data, the full texts were reviewed in detail, and no imputation of missing data was performed.

## 3 Results

### 3.1 Study selection

**FIGURE 1:**
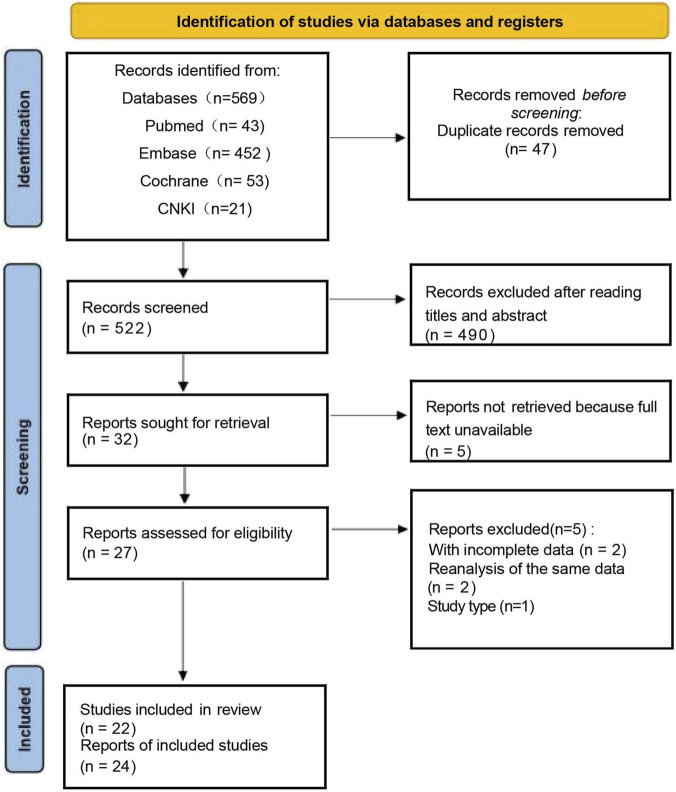
PRISMA flow diagram of study selection. Flow chart showing the identification, screening, eligibility assessment, and inclusion of studies in this systematic review and meta-analysis. A total of 569 records were identified through database searching, 522 records remained after duplicate removal, and 22 studies reported in 24 reports were ultimately included in the quantitative synthesis.

The process of study selection is illustrated in Figure 1. During evidence acquisition, 569 records were identified from PubMed, Embase, the Cochrane Library, and CNKI. A total of 522 records underwent title and abstract screening after 47 duplicates were removed, and 490 were excluded. Full texts were pursued for 32 reports, with 5 remaining unobtainable. Of the 27 reports assessed for eligibility, 5 were excluded for the following reasons: incomplete data (n = 2), reanalysis of previously reported data (n = 2), and inappropriate study design (n = 1) (Figure 1). Therefore, the systematic review and meta-analysis included 22 (Qiu et al., 2022; Chen Y. et al., 2025; Wang et al., 2023; Chen et al., 2024; Ma et al., 2024; Wei et al., 2024; Zhou et al., 2024; Chen Z. et al., 2025; Geng et al., 2025; Li et al., 2025; Wang H. et al., 2025; Wu et al., 2025; Yuan et al., 2025; Zhan et al., 2025; Zhang L. et al., 2025; Zhang M. et al., 2025; Zhao et al., 2025; Zhou et al., 2025; Li and Li, 2025; Pan et al., 2025; Wang J. et al., 2025; Zhao et al., 2026) studies spanning 24 reports.

### 3.2 Study characteristics

This review involved 22 (Qiu et al., 2022; Chen Y. et al., 2025; Wang et al., 2023; Chen et al., 2024; Ma et al., 2024; Wei et al., 2024; Zhou et al., 2024; Chen Z. et al., 2025; Geng et al., 2025; Li et al., 2025; Wang H. et al., 2025; Wu et al., 2025; Yuan et al., 2025; Zhan et al., 2025; Zhang L. et al., 2025; Zhang M. et al., 2025; Zhao et al., 2025; Zhou et al., 2025; Li and Li, 2025; Pan et al., 2025; Wang J. et al., 2025; Zhao et al., 2026) studies, all of which were conducted in China, enrolling adult patients aged 31–73 years (Table 1). This regional concentration of the available evidence should be considered when interpreting the external validity of the pooled findings. The types of surgical procedures ranged from liposuction to radical or modified mastectomy, lumbar interbody fusion, thoracoscopic lobectomy, gynecological laparoscopy, total hip or knee arthroplasties, laparoscopic cholecystectomy, Chiarian section and general surgery. Esketamine dosing differed across studies. The majority administered a loading dose and followed with intraoperative infusion (0.2–0.3 mg/kg/h), several more combined dexmedetomidine or postoperative patient-controlled analgesia. Control groups were given saline, dexmedetomidine alone or routine opioid-based anesthesia. Some studies assessed sleep outcomes on postoperative days 1, 2, 3 and day 7, whereas others reported only overall incidence. Sample sizes per group were between 30 and 130, with most studies showing even sex distribution. The operational definitions of PSD, sleep assessment instruments, cutoff values, individual sleep scores, and esketamine timing and route are summarized in Supplementary Table S2. Importantly, only studies that reported binary PSD event data, or provided event numbers based on their prespecified PSD definition, were included in the risk-ratio meta-analysis of PSD incidence; studies reporting only continuous sleep scores were not converted into dichotomous events. These features form the basis for the quantitative synthesis offered in the subsequent meta-analysis.

**TABLE 1:** General information of patients with incidence of PSD.

First author	Year	Age	Male/Female	Surgery type	Country	Group (with esketamine dosage)	Sleep disturbance (case)	Total
Chen, H	2024	33.3 ± 10.1	20/58	Liposuction surgery	China	Esketamine 0.3 mg/kg/h during induction, then 0.15 mg/kg/h intraoperatively + dexmedetomidine/remifentanil	NR	78
Chen, H	2024	33.1 ± 10.9	17/60	Liposuction surgery	China	Control: saline + dexmedetomidine/remifentanil	NR	77
Chen, Y	2025	48.30 ± 9.50	0/72	Modified radical mastectomy	China	Esketamine 0.2 mg/kg loading +0.1 mg/kg/h infusion	NR	72
Chen, Y	2025	49.60 ± 9.20	0/73	Modified radical mastectomy	China	Control (saline)	NR	73
Chen, Z	2025	NR	NR	Lumbar interbody fusion	China	Esketamine 0.2 mg/kg induction +0.02 mg/kg/h maintenance +1 mg/kg in PCIA for 48 h	POD1:13/39	39
Chen, Z	2025	NR	NR	Lumbar interbody fusion	China	Placebo (saline)	POD1: about 26/39	39
Geng, X	2025	50.44 ± 1.54	0/25	Radical mastectomy	China	Esketamine group (dose NR in extracted text)	POD3: 6/25	25
Geng, X	2025	54.00 ± 1.75	0/25	Radical mastectomy	China	Dexmedetomidine group	POD3: 5/25	25
Geng, X	2025	50.92 ± 1.53	0/25	Radical mastectomy	China	Esketamine + dexmedetomidine group	POD3: 11/25	25
Geng, X	2025	50.80 ± 1.29	0/25	Radical mastectomy	China	Control group	POD3: 14/25	25
Li, D	2025	70.42 ± 5.12	28/24	Thoracoscopic lobectomy	China	Esketamine 0.2 mg/kg IV bolus +5 μg/(kg·min) infusion to end of surgery	NR	52
Li, D	2025	69.68 ± 4.71	30/22	Thoracoscopic lobectomy	China	Control (saline)	NR	52
Li, X.-Y	2025	52 (47–58)	13/52	Laparoscopic abdominal	China	Esketamine 0.3 mg/kg/h intraoperative infusion	POD1: 28/65 POD3: 24/65; POD7: 29/65	65
Li, X.-Y	2025	51 (36.5–54.5)	9/56	Laparoscopic abdominal	China	Control (saline)	POD1: 42/65 POD3: 31/65 POD7: 37/65	65
Ma, C.-B	2024	NR	NR	THA/TKA	China	Group E: 0.20 mg/kg loading +0.125 mg/kg/h infusion +0.5 mg/kg for postoperative analgesia	NR	130
Ma, C.-B	2024	NR	NR	THA/TKA	China	Group P: placebo (saline)	NR	130
Pan, T	2025	55.36 ± 6.12	0/40	Gynecological laparoscopy	China	Esketamine + dexmedetomidine (both intraoperative continuous infusion)	10/40	40
Pan, T	2025	55.48 ± 6.23	0/40	Gynecological laparoscopy	China	Esketamine alone (intraoperative continuous infusion)	18/40	40
Pan, T	2025	55.39 ± 6.58	0/40	Gynecological laparoscopy	China	Control (saline)	22/40	40
Qiu, D	2022	43 (32–49)	0/92	Gynecological laparoscopy	China	Esketamine 0.3 mg/kg/h intraoperative infusion	POD1: 21/92; POD3: 7/92	92
Qiu, D	2022	45 (35–49)	0/91	Gynecological laparoscopy	China	Control (saline)	POD1: 40/91); POD3: 18/91	91
Wang, H	2025	73.02 ± 5.61	67/33	Elderly laparoscopic abdominal surgery	China	Intravenous esketamine (dose NR in extracted text)	POD1: 30/100; POD3: 20/100	100
Wang, H	2025	72.65 ± 5.33	56/44	Elderly laparoscopic abdominal surgery	China	Control (saline)	POD1: 45/100; POD3: 35/100	100
Wang, J	2025	45.3 ± 15.0	22/31	Ambulatory laparoscopic cholecystectomy	China	Esketamine 0.3 mg/(kg·h) intraoperative infusion	POD1: 12/53; POD3: 1/53	53
Wang, J	2025	46.9 ± 13.5	24/28	Ambulatory laparoscopic cholecystectomy	China	Control (saline)	POD1: 24/52; POD3: 4/52	52
Wang, P	2023	45.1 ± 10.9	4/26	Thyroidectomy/thyroidectomy	China	Esketamine 0.5 mg/kg bolus +0.24 mg/kg/h infusion	NR	30
Wang, P	2023	42.4 ± 12.0	5/25	Thyroidectomy/thyroidectomy	China	Control (0.9% NaCl)	NR	30
Wei, Q	2024	54 (46–63)	27/11	Total hip replacement	China	Es-D: esketamine + dexmedetomidine in PCA	NR	38
Wei, Q	2024	51 (39–61)	24/10	Total hip replacement	China	F-D: fentanyl + dexmedetomidine in PCA	NR	34
Wu, Y	2025	47.9 ± 9.4	23/20	Laparoscopic cholecystectomy	China	Esketamine 0.5 mg/kg/h intraoperative infusion	POD1: 25/43; POD2: 5/43 POD3: 1/43	43
Wu, Y	2025	49.7 ± 15.1	21/22	Laparoscopic cholecystectomy	China	Placebo (saline)	POD1: 35/43; POD2: 19/43; POD3: 8/43	43
Yuan, B	2025	49.7 ± 4.9	0/46	Gynecological	China	Experimental: esketamine loading 0.15 mg/kg + maintenance 0.15 mg/kg/h	NR	46
Yuan, B	2025	49.8 ± 4.5	0/46)	Gynecological	China	Experimental: esketamine loading 0.3 mg/kg + maintenance 0.3 mg/kg/h	NR	46
Yuan, B	2025	50.1 ± 5.2	0/46	Gynecological	China	Control: saline 5 mL + saline infusion 10 mL/h	NR	46
Zhan, Y	2025	48 (43–55)	0/58	Breast cancer surgery	China	Esketamine 0.2 mg/kg induction +0.4 mg/kg/h maintenance	POD1:16/58	58
Zhan, Y	2025	50 (43–52)	0/58	Breast cancer surgery	China	Control (placebo)	POD1:30/58	58
Zhang, L	2025	NR	0/35	Cesarean section	China	Esketamine in PCIA	NR	35
Zhang, L	2025	NR	0/35	Cesarean section	China	Esketamine + dexmedetomidine in PCIA	NR	35
Zhang, L	2025	NR	0/35	Cesarean section	China	Control PCIA	NR	35
Zhang, M	2025	44.00 ± 12.327	49/26	General surgery	China	Experimental: continuous intraoperative esketamine 0.3 mg/kg/h	POD1: 19/75; POD3: 14/75	75
Zhang, M	2025	43.97 ± 12.559	50/25	General surgeryapproach 73.3%	China	Control: equivalent saline	POD1: 37/75; POD3: 27/75	75
Zhao, H	2025	66 (61–70)	33/35	Thoracoscopic lung surgery	China	Esketamine 0.25 mg/kg loading +0.25 mg/kg/h infusion	POD1: 16/68; POD3: 8/68	68
Zhao, H	2025	68 (62–71)	31/37	Thoracoscopic lung surgery	China	Control (saline)	POD1: 30/68; POD3: 14/68	68
Zhao, Y	2025	70 ± 4.0	18/39	THA/TKA	China	Esketamine 0.72 mg/kg in PCIA + sufentanil	POD1: 16/57; POD2: 12/57; POD3: 4/57; POD7: 2/57	57
Zhao, Y	2025	70 ± 5.1	15/40	THA/TKA	China	Control PCIA (sufentanil only)	POD1: 31/55; POD2: 23/55 POD3: 12/55; POD7: 4/55	55
Zhou, Y	2024	47.8 ± 2.9	0/40	Radical mastectomy	China	Esketamine 0.3 mg/(kg·h) intraoperative infusion	POD1: 8/40; POD3: 5/40	40
Zhou, Y	2024	47.6 ± 4.8	0/40	Radical mastectomy	China	Control (saline)	POD1: 19/40; POD3: 14/40	40
Zhou, Y.-H	2025	31 (28, 36)	0/49)	Gynecologic	China	Experimental: esketamine 1 mg/kg/h for 30 min	POD1:8/49 POD3:5/49	49
Zhou, Y.-H	2025	31 (28, 37)	0/49	Gynecologic	China	Control: equivalent saline	POD1:17/49 POD3:14/49	49

Abbreviations: POD, postoperative day; THA, total hip arthroplasty; TKA, total knee arthroplasty; PCIA, patient-controlled intravenous analgesia; AIS, athens insomnia scale; NRS, numeric rating scale; PSQI, pittsburgh sleep quality index; RCSQ, Richards-Campbell Sleep Questionnaire; PSD, postoperative sleep disturbance.

### 3.3 The methodological quality of the included studies

**FIGURE 2:**
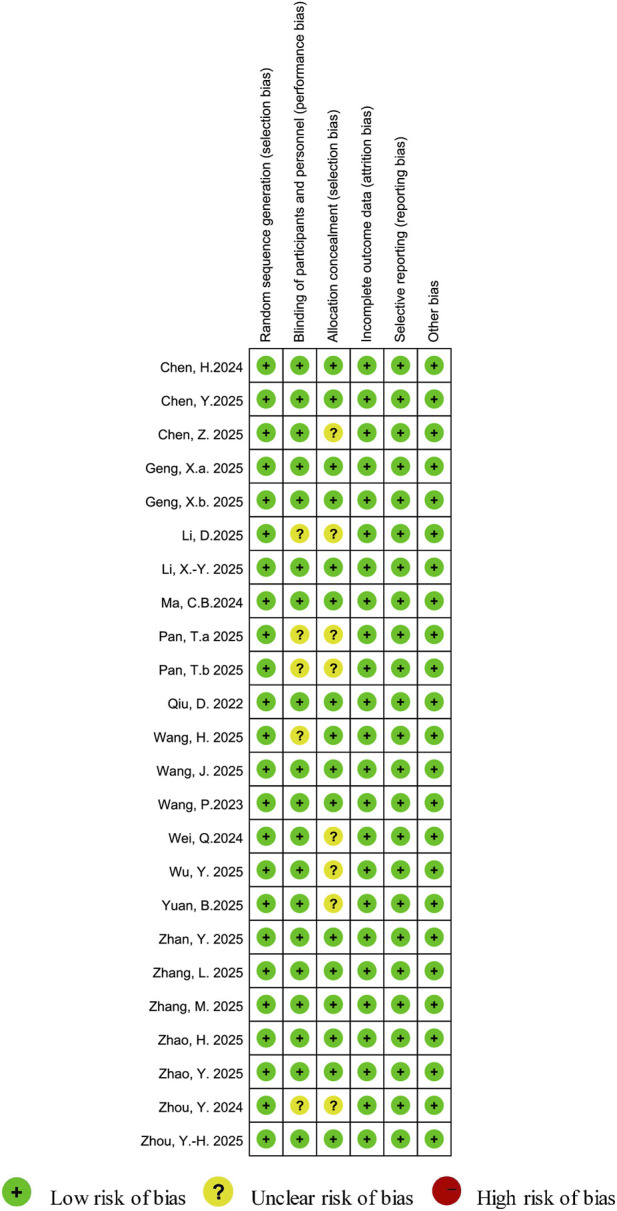
Risk-of-bias summary of the included randomized controlled trials. Summary of the authors’ judgments regarding risk of bias for each included study across the following domains: random sequence generation, allocation concealment, blinding of participants and personnel, blinding of outcome assessment, incomplete outcome data, selective reporting, and other bias.

**FIGURE 3:**
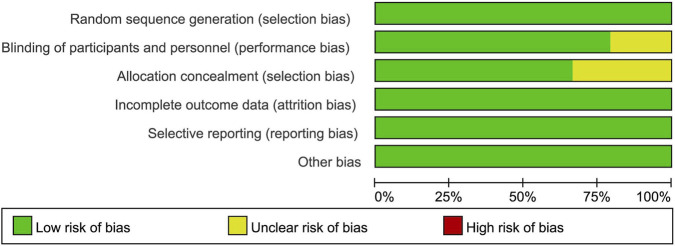
Risk-of-bias graph of the included randomized controlled trials. Graph showing the proportion of studies judged to be at low, unclear, or high risk of bias for each methodological domain assessed using the Cochrane Risk of Bias tool (ROB 1).

The methodological quality of the 22 (Qiu et al., 2022; Chen Y. et al., 2025; Wang et al., 2023; Chen et al., 2024; Ma et al., 2024; Wei et al., 2024; Zhou et al., 2024; Chen Z. et al., 2025; Geng et al., 2025; Li et al., 2025; Wang H. et al., 2025; Wu et al., 2025; Yuan et al., 2025; Zhan et al., 2025; Zhang L. et al., 2025; Zhang M. et al., 2025; Zhao et al., 2025; Zhou et al., 2025; Li and Li, 2025; Pan et al., 2025; Wang J. et al., 2025; Zhao et al., 2026) included studies was generally acceptable, and a summary of the risk-of-bias assessment is presented in Figures 2, 3. In most trials, the risk of bias was judged to be low in major areas, such as random sequence generation, incomplete outcome data, selective reporting, and other potential sources of bias. Randomization methods were clearly described, with some studies using computer-generated sequences and others using random number tables. Although most trials reported blinding of participants and personnel, this was unclear in a few studies, resulting in an unclear risk of performance bias. The risk of selection bias was also unclear in some studies because allocation concealment was not adequately reported.

Overall, outcome data were mostly complete with little attrition and all predefined outcomes were reported. Figure 2 displays the risk-of-bias assessment for each study, while Figure 3 shows the proportion of studies rated as low, unclear, or high risk across each domain. Although results suggest that the studies were reasonably credentialed about methodology, care should be taken when interpreting pooled findings given concerns regarding unclear risk in certain domains.

### 3.4 Quality of evidence

The quality of evidence for esketamine in the outcome measures (PSD, perioperative adverse events) was determined using the GRADE framework and is summarized in Table 2. Evidence was of moderate quality for PSD on postoperative days 1, 2, and 3 as all included studies were randomised trials but most outcomes were downgraded by one level owing to unclear allocation concealment. Esketamine significantly reduced the incidence of PSD compared to control groups, with relative risks between 0.40 and 0.58 and absolute reductions of 134–257 fewer cases per 1,000 patients. Evidence for PSD on POD7 was also rated moderate quality, although the limited number of events led to wider confidence intervals (RR 0.77, 95% CI 0.55–1.08). Regarding adverse events, high-quality evidence supported the safety profile for bradycardia. Headache, nightmares, and hypertension were supported by low-to-moderate quality evidence, while dizziness was rated very low due to few events, imprecision, and moderate-to-high heterogeneity (I^2^). Overall, the GRADE assessment indicates that esketamine may provide a meaningful reduction in early PSD incidence. However, the certainty of evidence for several adverse events was low or very low, particularly for dizziness, and the available safety data were insufficient to rule out increased risks of hemodynamic or neuropsychiatric adverse events.

**TABLE 2:** GRADE summary of efficacy of esketamine.

Quality assessment							No of patients		Effect		Quality	Importance
No of studies	Design	Risk of bias	Inconsistency	Indirectness	Imprecision	Other considerations	Esketamine	Control	Relative (95% CI)	Absolute		
PSD incidence-POD1												
12	Randomised trials	serious^1^	No serious inconsistency	No serious indirectness	No serious imprecision	None	212/739 (28.7%)	376/735 (51.2%)	RR 0.58 (0.51–0.66)	215 fewer per 1,000 (from 174 fewer to 251 fewer)	⊕⊕⊕Ο MODERATE	CRITICAL
							​	48.4%		203 fewer per 1,000 (from 165 fewer to 237 fewer)		
PSD incidence-POD2												
2	Randomised trials	serious^1^	No serious inconsistency	No serious indirectness	serious^2^	Strong association^3^	17/100 (17%)	42/98 (42.9%)	RR 0.4 (0.21–0.74)	257 fewer per 1,000 (from 111 fewer to 339 fewer)	⊕⊕⊕Ο MODERATE	CRITICAL
							​	43%		258 fewer per 1,000 (from 112 fewer to 340 fewer)		
PSD incidence-POD3												
12	Randomised trials	serious^1^	No serious inconsistency	No serious indirectness	No serious imprecision	None	107/692 (15.5%)	205/688 (29.8%)	RR 0.55 (0.45–0.68)	134 fewer per 1,000 (from 95 fewer to 164 fewer)	⊕⊕⊕Ο MODERATE	CRITICAL
							​	31.8%		143 fewer per 1,000 (from 102 fewer to 175 fewer)		
PSD incidence-POD7												
2	Randomised trials	No serious risk of bias	No serious inconsistency	No serious indirectness	serious^2^	None	31/122 (25.4%)	41/120 (34.2%)	RR 0.77 (0.55–1.08)	79 fewer per 1,000 (from 154 fewer to 27 more)	⊕⊕⊕Ο MODERATE	CRITICAL
							​	32.1%		74 fewer per 1,000 (from 144 fewer to 26 more)		
PONV												
11	Randomised trials	serious^1^	No serious inconsistency	No serious indirectness	No serious imprecision	None	120/700 (17.1%)	128/696 (18.4%)	RR 0.77 (0.54–1.11)	11 fewer per 1,000 (from 44 fewer to 31 more)	⊕⊕⊕Ο MODERATE	IMPORTANT
							​	17.4%		10 fewer per 1,000 (from 42 fewer to 30 more)		
Bradycardia												
3	Randomised trials	No serious risk of bias	No serious inconsistency	No serious indirectness	serious^1^	Strong association^2^	7/166 (4.2%)	24/165 (14.5%)	RR 0.28 (0.05–1.59)	105 fewer per 1,000 (from 138 fewer to 86 more)	⊕⊕⊕⊕ HIGH	IMPORTANT
							​	14.3%		103 fewer per 1,000 (from 136 fewer to 84 more)		
Headache												
3	Randomised trials	serious^1^	No serious inconsistency	No serious indirectness	serious^2^	None	16/217 (7.4%)	13/216 (6%)	RR 1.23 (0.61–2.47)	14 more per 1,000 (from 23 fewer to 88 more)	⊕⊕ΟΟ LOW	IMPORTANT
							​	5.1%		12 more per 1,000 (from 20 fewer to 75 more)		
Nightmare												
4	Randomised trials	serious^1^	No serious inconsistency	No serious indirectness	serious^2^	None	12/292 (4.1%)	13/287 (4.5%)	RR 0.82 (0.38–1.77)	8 fewer per 1,000 (from 28 fewer to 35 more)	⊕⊕ΟΟ LOW	IMPORTANT
							​	6.2%		11 fewer per 1,000 (from 38 fewer to 48 more)		
Dizziness												
8	Randomized trials	serious^1^	serious^2^	No serious indirectness	serious^3^	None	88/439 (20%)	62/433 (14.3%)	RR 1.16 (0.66–2.06)	23 more per 1,000 (from 49 fewer to 152 more)	⊕ΟΟΟ VERY LOW	IMPORTANT
							​	10.2%		16 more per 1,000 (from 35 fewer to 108 more)		
Hypertension												
3	Randomised trials	serious^1^	No serious inconsistency	No serious indirectness	serious^2^	None	17/131 (13%)	13/131 (9.9%)	RR 1.23 (0.47–3.19)	23 more per 1,000 (from 53 fewer to 217 more)	⊕⊕ΟΟ LOW	IMPORTANT
							​	10.2%		23 more per 1,000 (from 54 fewer to 223 more)		

1 downgraded for unclear allocation concealment across included trials.

2 Total number of events is less than 300.

3 RR < 0.5.

4 downgraded for unclear allocation concealment across included trials.

5 Total number of events is less than 300.

6 RR < 0.2.

7 downgraded for unclear allocation concealment across included trials.

8 Total number of events is less than 300.

9 downgraded for unclear allocation concealment across included trials.

10 Total number of events is less than 300.

11 downgraded for unclear allocation concealment across included trials.

12 I2 values indicated moderate or high thresholds for statistical heterogeneity.

13 Total number of events is less than 300.

14 downgraded for unclear allocation concealment across included trials.

15 Total number of events is less than 300.

Abbreviations: RR, risk ratio; CI, confidence interval; I^2^, inconsistency index; GRADE, grading of recommendations assessment, Development and Evaluation; RCT, randomized controlled trial.

### 3.5 Results of meta-analysis

#### 3.5.1 Meta-analysis of PSD at different time points

##### 3.5.1.1 Sleep disturbance-POD1

**FIGURE 4:**
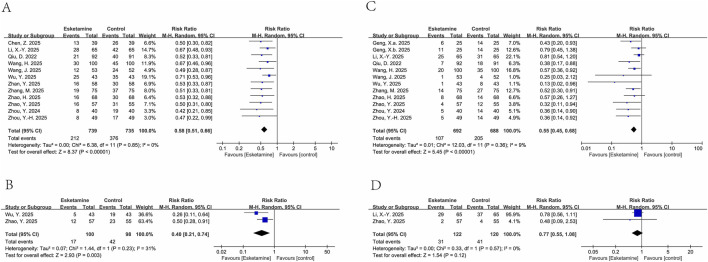
Forest plots of postoperative sleep disturbance at different postoperative time points. **(A)** Postoperative day 1 (POD1). **(B)** Postoperative day 2 (POD2). **(C)** Postoperative day 3 (POD3). **(D)** Postoperative day 7 (POD7). Forest plots compare perioperative esketamine with control for the incidence of postoperative sleep disturbance. Pooled effect sizes are presented as risk ratios (RRs) with 95% confidence intervals (CIs).

**FIGURE 5:**
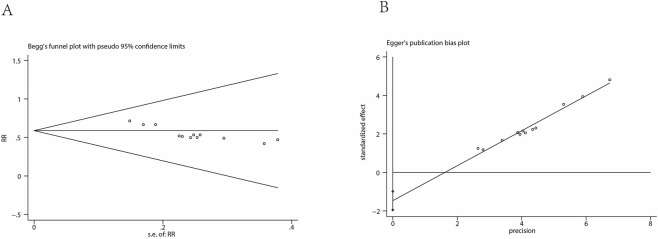
Funnel plot for publication bias assessment of postoperative sleep disturbance on postoperative day 1. Funnel plot used to evaluate potential publication bias or small-study effects for the primary outcome of postoperative sleep disturbance on POD1. **(A)** Begg. **(B)** Egger.

On POD1, the incidence of PSD was reported in 12 (Qiu et al., 2022; Zhou et al., 2024; Chen Z. et al., 2025; Li et al., 2025; Wang H. et al., 2025; Wu et al., 2025; Zhan et al., 2025; Zhang M. et al., 2025; Zhao et al., 2025; Zhou et al., 2025; Wang J. et al., 2025; Zhao et al., 2026) trials involving 1,474 patients. The pooled analysis found that perioperative esketamine significantly reduced the risk of sleep disturbance compared with the control group (RR = 0.58, 95% CI: 0.51–0.66), with low observed statistical heterogeneity among the included studies (I^2^ = 0%, P = 0.85) (Figure 4A). This I^2^ value reflects the degree of statistical inconsistency in the pooled binary outcome and should not be interpreted as evidence of complete clinical homogeneity across trials. Given the subjective and multifactorial nature of PSD, this finding was interpreted cautiously and was further considered in the Discussion together with possible publication bias, outcome definition, and regional concentration of the evidence. However, Begg’s test (P = 0.028) and Egger’s test (P < 0.001) both suggested potential publication bias or small-study effects (Figure 5). As a result, the pooled outcome should be interpreted cautiously. To further examine the possible effect of publication bias, a trim-and-fill analysis was carried out. No potentially missing studies were imputed, and the pooled effect size remained unchanged after adjustment (RR = 0.588, 95% CI: 0.461–0.716), indicating that the overall result was robust despite the asymmetry suggested by Begg’s and Egger’s tests.

##### 3.5.1.2 Sleep disturbance-POD2

On POD2, two trials (Wu et al., 2025; Zhao et al., 2026) evaluated the incidence of PSD. The pooled results showed that esketamine was associated with a significantly lower risk of sleep disturbance than the control group (RR = 0.40, 95% CI: 0.28–0.58), with low observed statistical heterogeneity (I^2^ = 0%). However, this result was based on only two trials and the I^2^ statistic had limited ability to detect between-study variability. Therefore, the POD2 result should be interpreted cautiously. Further details are presented in Figure 4B.

##### 3.5.1.3 Sleep disturbance-POD3

On POD3, 11 (Qiu et al., 2022; Zhou et al., 2024; Geng et al., 2025; Li et al., 2025; Wang H. et al., 2025; Wu et al., 2025; Zhang M. et al., 2025; Zhao et al., 2025; Zhou et al., 2025; Wang J. et al., 2025; Zhao et al., 2026) trials involving 1,380 patients assessed the incidence of PSD. The pooled analysis found that esketamine significantly reduced the incidence of sleep disturbance compared with the control group (RR = 0.55, 95% CI: 0.45–0.68), with low heterogeneity across the included studies (I^2^ = 9%, P = 0.36), as shown in Figure 4C.

##### 3.5.1.4 Sleep disturbance-POD7

On POD7, two trials (Li et al., 2025; Zhao et al., 2026) involving 242 patients reported the incidence of PSD. The pooled analysis revealed no significant difference between the esketamine and control groups (RR = 0.77, 95% CI: 0.55–1.08). These findings indicate that the beneficial effect of esketamine on PSD may be more evident shortly after surgery than at POD7 (Figure 4D).

#### 3.5.2 Subgroup analysis

##### 3.5.2.1 Subgroup analysis by loading/bolus administration on POD1

**FIGURE 6:**
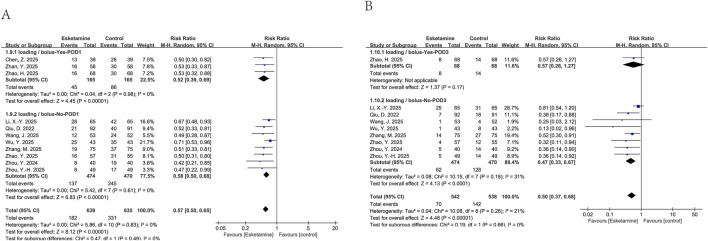
Subgroup analyses according to loading or bolus administration of esketamine. **(A)** Subgroup analysis of postoperative sleep disturbance on POD1 according to whether a loading or bolus dose was administered. **(B)** Subgroup analysis of postoperative sleep disturbance on POD3 according to whether a loading or bolus dose was administered. Pooled effect sizes are expressed as RRs with 95% CIs.

Subgroup analysis according to whether a loading or bolus dose was administered showed that esketamine was associated with a lower incidence of PSD on POD1 in both subgroups. In studies using a loading or bolus regimen, the pooled analysis of 3 trials (Chen Z. et al., 2025; Zhan et al., 2025; Zhao et al., 2025) showed a significant reduction in POD1 sleep disturbance (RR = 0.52, 95% CI: 0.39–0.69). A similar result was observed in the non-loading/bolus subgroup, in which 8 trials (Qiu et al., 2022; Zhou et al., 2024; Li et al., 2025; Wu et al., 2025; Zhang M. et al., 2025; Zhou et al., 2025; Wang J. et al., 2025; Zhao et al., 2026) also favored esketamine (RR = 0.58, 95% CI: 0.50–0.68). No significant subgroup difference was detected between the two strategies (P = 0.49) (Figure 6A).

##### 3.5.2.2 Subgroup analysis by loading/bolus administration on POD3

Subgroup analysis based on whether a loading or bolus dose was used showed a numerically lower incidence of PSD on POD3 in both subgroups. In the loading/bolus subgroup, the pooled estimate from 1 trial (Zhao et al., 2025) suggested a reduction in POD3 sleep disturbance, although the difference was not statistically significant (RR = 0.57, 95% CI: 0.26–1.27). In the non-loading/bolus subgroup, pooled analysis of 8 trials (Qiu et al., 2022; Zhou et al., 2024; Li et al., 2025; Wu et al., 2025; Zhang M. et al., 2025; Zhou et al., 2025; Wang J. et al., 2025; Zhao et al., 2026) showed a significant benefit in favor of esketamine (RR = 0.47, 95% CI: 0.33–0.67). No significant subgroup difference was observed (P = 0.66) (Figure 6B).

##### 3.5.2.3 Subgroup analysis according to preoperative sleep disorder on POD1

**FIGURE 7:**
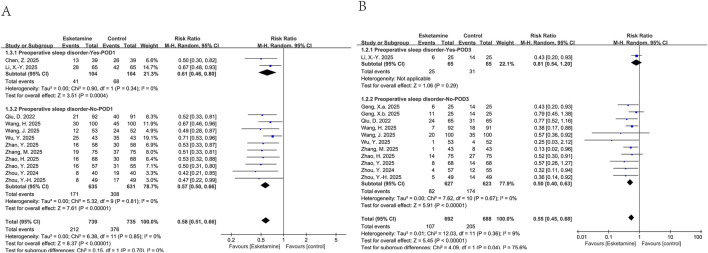
Subgroup analyses according to preoperative sleep status. **(A)** Subgroup analysis of postoperative sleep disturbance on POD1 stratified by the presence or absence of preoperative sleep disturbance. **(B)** Subgroup analysis of postoperative sleep disturbance on POD3 stratified by the presence or absence of preoperative sleep disturbance. Pooled effect sizes are expressed as RRs with 95% CIs.

Subgroup analysis according to the presence of preoperative sleep disorder showed that esketamine significantly reduced the incidence of PSD on POD1 in both subgroups. In patients with preoperative sleep disorder, pooled analysis of 2 trials (Chen Z. et al., 2025; Li et al., 2025) showed a significant reduction in POD1 sleep disturbance (RR = 0.61, 95% CI: 0.46–0.80). A similar effect was observed in patients without preoperative sleep disorder, based on 10 trials (Qiu et al., 2022; Zhou et al., 2024; Wang H. et al., 2025; Wu et al., 2025; Zhan et al., 2025; Zhang M. et al., 2025; Zhao et al., 2025; Zhou et al., 2025; Wang J. et al., 2025; Zhao et al., 2026) (RR = 0.57, 95% CI: 0.50–0.66). No significant subgroup difference was detected (P = 0.70) (Figure 7A).

##### 3.5.2.4 Subgroup analysis according to preoperative sleep disorder on POD3

Subgroup analysis on POD3 showed different results between the two subgroups. In patients with preoperative sleep disorder, one trial (Li et al., 2025) did not show a significant reduction in PSD with esketamine (RR = 0.81, 95% CI: 0.54–1.20). By contrast, in patients without preoperative sleep disorder, pooled analysis of 10 trials (Qiu et al., 2022; Zhou et al., 2024; Geng et al., 2025; Wang H. et al., 2025; Wu et al., 2025; Zhang M. et al., 2025; Zhao et al., 2025; Zhou et al., 2025; Wang J. et al., 2025; Zhao et al., 2026) demonstrated a significant benefit in favor of esketamine (RR = 0.50, 95% CI: 0.40–0.63). A significant subgroup difference was observed on POD3 (P = 0.04) (Figure 7B).

##### 3.5.2.5 Subgroup analysis according to timing of administration on POD1

**FIGURE 8:**
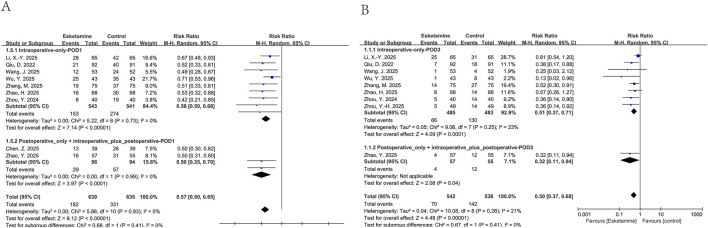
Subgroup analyses according to the timing of esketamine administration. **(A)** Subgroup analysis of postoperative sleep disturbance on POD1 comparing intraoperative-only administration with postoperative-only or intraoperative plus postoperative administration. **(B)** Subgroup analysis of postoperative sleep disturbance on POD3 according to timing of esketamine administration. Pooled effect sizes are expressed as RRs with 95% CIs.

Subgroup analysis according to the timing of esketamine administration showed that esketamine significantly reduced the incidence of PSD on POD1 in both subgroups. In studies using intraoperative administration only, pooled analysis of 7 (Qiu et al., 2022; Li et al., 2025; Wu et al., 2025; Zhang M. et al., 2025; Zhao et al., 2025; Wang J. et al., 2025; Zhao et al., 2026) trials showed a significant reduction in POD1 sleep disturbance (RR = 0.58, 95% CI: 0.50–0.68). A similar result was observed in studies using postoperative-only or intraoperative plus postoperative administration, based on 2 trials (Chen Y. et al., 2025; Zhao et al., 2026) (RR = 0.50, 95% CI: 0.35–0.70). No significant subgroup difference was detected (P = 0.41) (Figure 8A).

##### 3.5.2.6 Subgroup analysis according to timing of administration on POD3

Subgroup analysis on POD3 showed that esketamine was associated with a lower incidence of PSD in both subgroups. In the intraoperative-only subgroup, pooled analysis of 8 trials (Qiu et al., 2022; Zhou et al., 2024; Li et al., 2025; Wu et al., 2025; Zhang M. et al., 2025; Zhao et al., 2025; Zhou et al., 2025; Wang J. et al., 2025) demonstrated a significant benefit in favor of esketamine (RR = 0.51, 95% CI: 0.37–0.71). In the postoperative-only or intraoperative plus postoperative subgroup, the available trial (Zhao et al., 2026) also showed a significant reduction in POD3 sleep disturbance (RR = 0.32, 95% CI: 0.11–0.94). No significant subgroup difference was observed between the two subgroups (P = 0.41) (Figure 8B).

##### 3.5.2.7 Subgroup analysis according to maintenance infusion rate on POD1

**FIGURE 9:**
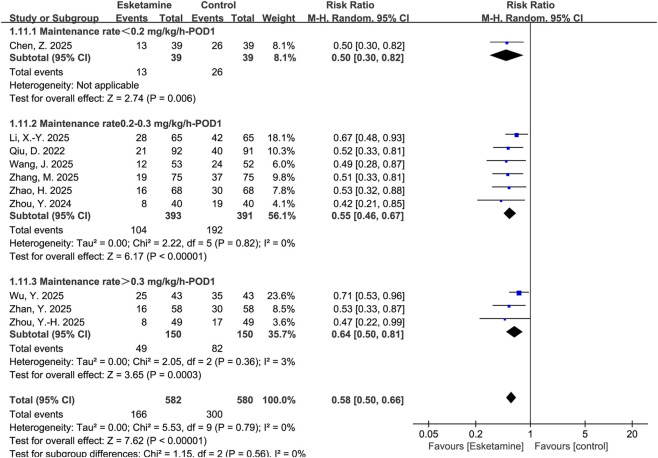
Subgroup analysis according to maintenance infusion rate on postoperative day 1. Forest plot showing the effect of perioperative esketamine on postoperative sleep disturbance on POD1 stratified by maintenance infusion rate (<0.2 mg/kg/h, 0.2–0.3 mg/kg/h, and >0.3 mg/kg/h). Pooled effect sizes are expressed as RRs with 95% CIs.

According to subgroup analysis based on maintenance infusion rate, esketamine significantly lowered the incidence of PSD on POD1 across all dose categories. In the subgroup with a maintenance rate of <0.2 mg/kg/h, the included trial (Chen Z. et al., 2025) showed a significant reduction in sleep disturbance (RR = 0.50, 95% CI: 0.30–0.82). In studies using a maintenance rate of 0.2–0.3 mg/kg/h, pooled analysis of six trials (Qiu et al., 2022; Li et al., 2025; Zhang M. et al., 2025; Zhao et al., 2025; Wang J. et al., 2025; Zhao et al., 2026) likewise demonstrated a significant advantage for esketamine (RR = 0.55, 95% CI: 0.46–0.67). A similar benefit was found in the >0.3 mg/kg/h subgroup, based on three trials (Wu et al., 2025; Zhan et al., 2025; Zhou et al., 2025) (RR = 0.64, 95% CI: 0.50–0.81). No significant difference was detected among the three dose categories (P = 0.56) (Figure 9).

#### 3.5.3 Secondary outcomes

##### 3.5.3.1 Bradycardia, dizziness, and headache

**FIGURE 10:**
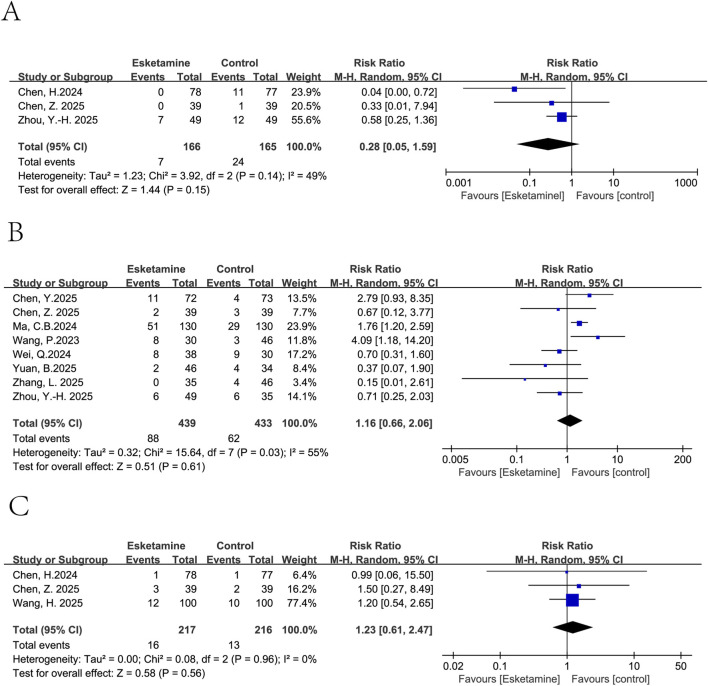
Forest plots of selected adverse events associated with perioperative esketamine. **(A)** Bradycardia. **(B)** Dizziness. **(C)** Headache. Forest plots compare perioperative esketamine with control for each adverse event, with pooled effect sizes presented as RRs and 95% CIs.

Bradycardia was reported in three trials (Chen et al., 2024; Chen Z. et al., 2025; Zhou et al., 2025) involving 331 patients, and pooled analysis found no significant difference between the esketamine and control groups (RR = 0.28, 95% CI: 0.05–1.59), with moderate heterogeneity across studies (I^2^ = 49%, P = 0.14). Eight trials (Chen Y. et al., 2025; Wang et al., 2023; Ma et al., 2024; Wei et al., 2024; Chen Z. et al., 2025; Yuan et al., 2025; Zhang L. et al., 2025; Zhou et al., 2025) including 872 patients assessed dizziness, and the pooled results also showed no statistically significant difference between groups (RR = 1.16, 95% CI: 0.66–2.06), although moderate heterogeneity was present (I^2^ = 55%, P = 0.03), because the confidence interval was wide and crossed the null effect, this result does not exclude a possible increase in dizziness risk. Headache was evaluated in three trials (Chen et al., 2024; Chen Z. et al., 2025; Wang H. et al., 2025) involving 433 patients, and no significant difference was observed between the esketamine and control groups (RR = 1.23, 95% CI: 0.61–2.47), with no heterogeneity detected (I^2^ = 0%, P = 0.96) (Figure 10).

##### 3.5.3.2 Hypertension, nightmare, and PONV

**FIGURE 11:**
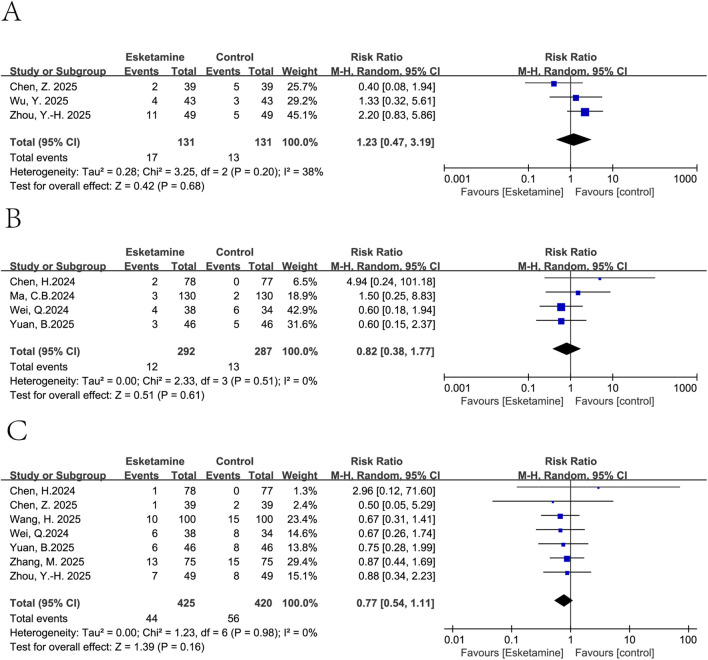
Forest plots of additional adverse events associated with perioperative esketamine. **(A)** Hypertension. **(B)** Nightmare. **(C)** Postoperative nausea and vomiting (PONV). Forest plots compare perioperative esketamine with control for each adverse event, with pooled effect sizes presented as RRs and 95% CIs.

Three trials (Chen Z. et al., 2025; Wu et al., 2025; Zhou et al., 2025) involving 262 patients evaluated hypertension, and no significant difference was found between the esketamine and control groups (RR = 1.23, 95% CI: 0.47–3.19), with low-to-moderate heterogeneity (I^2^ = 38%, P = 0.20). However, this analysis was based on few studies and had a wide confidence interval; therefore, an increased hemodynamic risk cannot be ruled out. Four trials (Chen et al., 2024; Ma et al., 2024; Wei et al., 2024; Yuan et al., 2025) including 579 patients assessed nightmare, and the pooled analysis also showed no statistically significant difference (RR = 0.82, 95% CI: 0.38–1.77), with no heterogeneity (I^2^ = 0%, P = 0.51). Furthermore, seven trials (Chen et al., 2024; Wei et al., 2024; Chen Z. et al., 2025; Wang H. et al., 2025; Yuan et al., 2025; Zhang M. et al., 2025; Zhou et al., 2025) involving 845 patients reported PONV, and the incidence did not differ significantly between groups (RR = 0.77, 95% CI: 0.54–1.11), with no evidence of heterogeneity (I^2^ = 0%, P = 0.98) (Figure 11).

### 3.6 Trial sequential analysis

**FIGURE 12:**
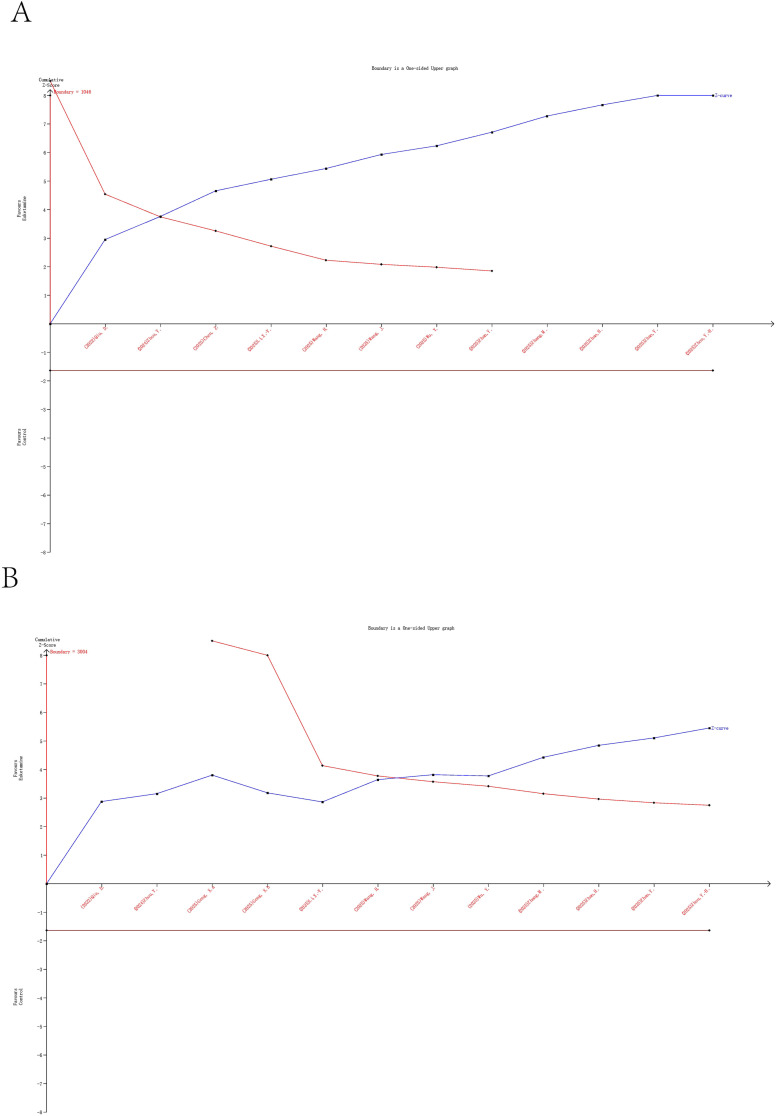
Trial sequential analyses of postoperative sleep disturbance. **(A)** Trial sequential analysis (TSA) for postoperative sleep disturbance on POD1. **(B)** TSA for postoperative sleep disturbance on POD3. The cumulative Z-curve is shown in relation to the conventional significance boundary, trial sequential monitoring boundary, and required information size.

PSD on POD1 and POD3 was analyzed using trial sequential analysis (TSA). The cumulative Z-curve on POD1 surpassed both the conventional significance and sequential monitoring boundaries, supporting the robustness of esketamine’s effect in reducing PSD. The analysis on POD3 showed a similar pattern, as the cumulative Z-curve crossed the monitoring boundary, supporting the adequacy of the existing evidence and a low probability of a random effect (Figure 12).

### 3.7 Sensitivity analysis

Sensitivity analyses using a leave-one-out approach were performed for PSD on POD1 and POD3. For POD1, the pooled effect estimate changed little after removing studies one at a time, and the overall result consistently favored esketamine, indicating good result stability. A similar pattern was observed for POD3, where sequential exclusion of individual studies produced only limited shifts in the pooled estimate without changing the overall direction of effect. Taken together, these findings suggest that the results for POD1 and POD3 were robust and were not dependent on any single study (Supplementary Figures S1,S2).

## 4 Discussion

### 4.1 Overview

PSD is clinically important because it has been associated with increased physical discomfort, psychological distress, impaired neurocognitive function, delayed postoperative recovery, and a greater risk of delirium (Yuan et al., 2024; He et al., 2022). The primary findings of this meta-analysis are outlined as follows. First, during the early postoperative period, perioperative esketamine significantly lowered the incidence of sleep disturbance, with clear benefits on POD1 through POD3, but not on POD7. Second, this beneficial effect was generally consistent across most subgroup analyses according to loading strategy, timing of administration, and maintenance infusion rate, although a significant subgroup difference was identified on POD3 when studies were stratified by preoperative sleep status. Third, no significant increase in the adverse events examined in this review was observed with perioperative esketamine, including bradycardia, dizziness, headache, hypertension, nightmares, and postoperative nausea and vomiting. Finally, the main findings appeared robust based on both sensitivity analysis and trial sequential analysis. Even so, potential publication bias in the POD1 analysis and unclear allocation concealment in several included studies should be considered in the interpretation of the findings.

### 4.2 Mechanistic basis and explanation for the early benefit

Our meta-analysis showed that perioperative esketamine was associated with a reduced incidence of PSD during POD1–3. This temporal pattern is clinically and biologically plausible, as sleep disruption is typically most severe in the first few nights after surgery, when pain, surgical stress, inflammatory responses, opioid exposure, circadian misalignment, and environmental disturbance interact to disrupt normal sleep architecture (Rampes et al., 2019; Lin et al., 2021). There are several potential mechanisms underlying this early benefit. First, esketamine may relieve central sensitization and improve postoperative analgesia while decreasing perioperative opioid consumption by blocking N-methyl-D-aspartate receptors, thus breaking the vicious circle of pain - > analgesic need - > sleep quality (Chen et al., 2024; Onen et al., 2005). Second, ketamine-linked agents can affect sleep more directly via modulation of slow-wave activity and neuroplasticity-related signaling pathways, including brain-derived neurotrophic factor, which has been shown to be involved in mood modulation and restoration of sleep architecture (Duncan and Zarate, 2013; Song and Zhu, 2021). The immediate postoperative period, when nociceptive and stress-related perturbations peak, is likely when these effects are most relevant. By POD7, however, postoperative sleep may be increasingly influenced by other determinants, such as functional recovery, wound healing, ward environment, and individual baseline susceptibility to sleep disturbance, which may help explain why the apparent benefit of esketamine was no longer statistically significant at that time point.

### 4.3 Subgroup analyses and safety

Subgroup analyses provided additional support for the robustness of the main findings. On POD1, the association between perioperative esketamine and a reduced incidence of PSD remained broadly consistent across subgroups defined by bolus/loading administration, timing of administration, and maintenance infusion rate, and no significant subgroup differences were observed in most comparisons. A comparable trend was also noted on POD3. However, a significant subgroup difference was identified when studies were stratified according to preoperative sleep status. This result should be interpreted with caution, as some subgroup categories were informed by only a small number of studies and therefore may have been underpowered; accordingly, it should be regarded as exploratory rather than confirmatory. With respect to safety, the pooled results of the PSD-focused trials did not show statistically significant increases in bradycardia, dizziness, headache, hypertension, nightmares, or postoperative nausea and vomiting. However, this finding should not be interpreted as definitive evidence of a neutral safety profile. Adverse-event analyses in the present review were limited by inconsistent reporting, small numbers of contributing trials for several outcomes, low event counts, and low or very low certainty of evidence for some safety endpoints. In particular, the analysis of dizziness showed moderate heterogeneity, and the certainty of evidence was rated as very low. Moreover, the absence of statistical significance in this review does not exclude clinically relevant risks, especially for dose-dependent or population-specific adverse events.

This cautious interpretation is important because recent broader perioperative esketamine meta-analyses have reported increased risks of certain adverse events. For example, Lu et al. found that esketamine increased dizziness (RR = 1.29, 95% CI: 1.03–1.62) and perioperative hypertension (RR = 2.40, 95% CI: 1.16–4.97) in elderly surgical patients (Lu et al., 2026). The discrepancy between our findings and these previous results may be explained by differences in target populations, outcome focus, included studies, definitions of adverse events, dosing regimens, timing of administration, background anesthesia/analgesia protocols, and statistical power. Therefore, perioperative esketamine should be used with appropriate monitoring, particularly for hemodynamic responses and neuropsychiatric symptoms such as dizziness, hallucinations, nightmares, and dissociation.

### 4.4 Comparison with previous studies

These results are largely consistent with recent trials indicating that perioperative esketamine could positively influence postoperative sleep. In women undergoing gynecological laparoscopy, Qiu et al. found that intraoperative esketamine infusion was associated with a lower incidence of PSD (Qiu et al., 2022). Similarly, Song et al. reported that a single low dose of esketamine administered during surgical abortion improved sleep on the first postoperative night in women with preoperative sleep disturbance (Song et al., 2025). Zhan et al. also demonstrated that subanesthetic esketamine improved postoperative sleep quality and reduced sleep disturbance after breast cancer surgery (Zhan et al., 2025). In contrast, Chen et al. did not detect a significant improvement in postoperative sleep quality with intraoperative subanesthetic esketamine in patients undergoing modified radical mastectomy (Chen Y. et al., 2025). The variation in findings across studies may be explained by differences in surgical technique, baseline sleep profiles, dosing regimens and timing of administration, background analgesia, and the definitions used for sleep-related outcomes. Within this context, the current meta-analysis contributes further evidence suggesting that the effect of perioperative esketamine is primarily limited to the early postoperative period, with the most evident benefit occurring between POD1 and POD3, while no statistically significant effect was observed on POD7. It should also be emphasized that the current evidence mainly supports a reduction in early binary PSD incidence rather than a comprehensive improvement in all sleep dimensions. Several recent trials have evaluated more specific sleep-related outcomes, including sleep quality scores, RCSQ scores, EEG-derived indicators, and actigraphy-based parameters such as total sleep time, sleep efficiency, and wake after sleep onset. However, these outcomes were not uniformly reported and could not be consistently pooled in the present meta-analysis. For example, one recent randomized trial in patients undergoing modified radical mastectomy did not show a significant improvement in postoperative sleep quality after intraoperative subanesthetic esketamine (Chen Y. et al., 2025), whereas other studies suggested potential improvements in early sleep quality or objective sleep parameters in selected surgical populations (Yuan et al., 2025; Zhan et al., 2025). Therefore, the available evidence should be interpreted as supporting a short-term reduction in PSD incidence, while the effects of esketamine on detailed sleep architecture, sleep continuity, and objective sleep recovery remain incompletely characterized. Another important consideration is that most trials contributing to the pooled incidence analysis relied on subjective sleep assessments. Although subjective scales are clinically meaningful and easy to implement, they may be influenced by postoperative pain, opioid consumption, anxiety, nausea, environmental disturbance, and patients’ expectations regarding recovery. Therefore, the observed reduction in PSD incidence may partly reflect improvements in pain control or overall postoperative comfort rather than a direct normalization of sleep architecture. This possibility is particularly relevant for esketamine, given its analgesic and opioid-sparing properties. Accordingly, the present findings should be interpreted as evidence of improved patient-reported early sleep disturbance, rather than definitive evidence that esketamine objectively restores postoperative sleep structure.

### 4.5 Clinical heterogeneity and interpretation of low statistical heterogeneity

An important method problem in this review is the explanation of the low I^2^ value of PSD observed on POD1 and POD2. Although statistical heterogeneity is very low, it should not be interpreted as a lack of clinical heterogeneity. PSD is a multi-factorial result, which is affected by the type of surgery, patient’s age, baseline sleep quality, postoperative pain, opioid consumption, inflammatory reaction, ward environment and perioperative analgesia strategy. In this review, the included tests involve various surgical procedures, including gynecological laparoscopy, breast surgery, abdominal surgery, orthopedic surgery, thoracic surgery, lumbar interbody fusion and outpatient laparoscopic cholecystectomy. Eschetamin regimen also varies greatly in terms of loading dose, maintenance infusion rate, administration time, and use in patient-controlled intravenous pain relief. Therefore, the low I^2^ value may indicate the consistency of the direction and magnitude of the summary binary effect estimation, rather than the real clinical consistency between the tests included.

In order to solve this problem, random effect models were used in all analyses, and subgroup analysis was carried out according to loading or suppository administration, preoperative sleep status, administration time and datad maintenance infusion rate. The omission sensitivity analysis further shows that the discovery of POD1 and POD3 is not driven by any single study. However, the residual clinical heterogeneity cannot be fully explored because data at the level of a single patient cannot be obtained, and some subgroups only contain a small number of studies. Therefore, the aggregated estimates should be regarded as a general summary of available random evidence, rather than as evidence that estamin has a uniform effect in all surgical populations.

### 4.6 Methodological considerations and robustness of the evidence

Methodologically speaking, the results of this meta-analysis seem relatively robust; however, there are some limitations to note. The primary outcome on POD1 showed potential publication bias or small-study effects according to Begg’s and Egger’s tests, which may have affected the asymmetry of the available evidence and the resulting pooled estimate. After trim-and-fill analysis detected no missing studies and the pooled effect estimate remained largely unchanged, it appears that publication bias had minimal impact on the primary conclusion. Nevertheless, because only a few studies were included and all originated from China, the risk of small-study effects persists, and the POD1 results should be treated with caution. Leave-one-out sensitivity analyses also supported the robustness of the findings: for both POD1 and POD3, sequential removal of individual studies had little impact on the pooled estimates and did not change the direction of effect, indicating that the observed benefit was not driven by any single trial. Moreover, the cumulative Z-curve crossed the monitoring boundary for POD1 and POD3 in trial sequential analysis, supporting the conclusion that the evidence represents a true effect rather than a chance finding due to limited data or multiple testing. However, the certainty of evidence should not be overstated. According to the GRADE assessment, the certainty of evidence for PSD on POD1, POD2, and POD3 was rated as moderate, with unclear allocation concealment being the most common reason for downgrading. For POD7, the evidence was downgraded because of imprecision. For adverse events, the certainty of evidence ranged from high to very low, with downgrading mainly attributable to imprecision, heterogeneity, and the limited number of studies for some outcomes. Taken together, these findings support the stability of the early postoperative sleep benefit associated with perioperative esketamine; Nonetheless, due to methodological limitations in the current evidence, further high-quality and adequately powered multicenter research is needed.

### 4.7 Limitations and suggestions for practice

There are a number of limitations in this meta-analysis. First, the geographic generalizability of the findings is limited. All included trials were conducted in China, indicating that the current evidence base is regionally concentrated. Perioperative practice may differ across countries and healthcare systems in terms of anesthesia protocols, postoperative analgesic regimens, opioid-sparing strategies, nursing care, discharge pathways, and availability of sleep assessment tools. Patient characteristics, cultural perceptions of sleep disturbance, and thresholds for reporting sleep-related symptoms may also vary across populations. Therefore, the pooled estimates should not be assumed to directly apply to all global surgical settings. Future multinational and multicenter randomized trials are needed to confirm the reproducibility and external validity of the observed early postoperative sleep benefit. Second, the definition and measurement of PSD varied across the included trials. Different studies used different instruments, including the Athens Insomnia Scale, Pittsburgh Sleep Quality Index, Numeric Rating Scale for sleep, Richards-Campbell Sleep Questionnaire, and actigraphy-based parameters. To reduce measurement bias, we did not transform continuous sleep scores into dichotomous PSD events; instead, the meta-analysis of PSD incidence was restricted to trials that reported binary event data according to the authors’ original definitions or cutoff values. Nevertheless, because the cutoff values and assessment tools were not fully uniform across trials, residual measurement heterogeneity remains possible. Future trials should adopt standardized and validated definitions of PSD and report both continuous sleep-scale scores and binary event data to improve comparability across studies. In addition, low statistical heterogeneity in some pooled analyses should not be equated with clinical homogeneity. The included trials differed in surgical type, patient age, baseline sleep condition, perioperative analgesic regimen, esketamine dose, timing of administration, and sleep assessment approach. Because aggregate data rather than individual patient-level data were available, the influence of these clinical factors could not be fully quantified. In particular, the I^2^ value of 0% for POD1 and POD2 should be interpreted cautiously because PSD is a subjective outcome and because statistical heterogeneity may be underestimated when the number of studies is small, when outcome definitions are relatively standardized, or when published evidence is regionally concentrated and potentially affected by small-study effects. Future trials and individual patient data meta-analyses are needed to determine whether the effect of esketamine differs across specific surgical populations and baseline sleep-risk profiles. Moreover, although the number of included RCTs appears substantial, the evidence base for objective and specific sleep parameters remains limited. Most pooled analyses focused on binary PSD incidence at early postoperative time points, and this incidence was largely derived from subjective sleep scales or patient-reported sleep quality. Subjective assessments may be confounded by postoperative pain intensity, opioid consumption, anxiety, nausea, ward environment, and patients’ expectations regarding recovery. Therefore, the observed reduction in PSD incidence may overestimate the direct effect of esketamine on sleep itself, particularly because esketamine may improve patient-reported sleep partly through analgesic and opioid-sparing effects. Detailed outcomes such as total sleep time, sleep efficiency, wake after sleep onset, REM/NREM sleep structure, polysomnography-derived sleep architecture, and EEG-derived sleep features were inconsistently reported and could not be quantitatively pooled. As a result, the present findings should not be interpreted as definitive evidence that esketamine objectively improves all dimensions of postoperative sleep. Future trials should incorporate objective sleep monitoring, such as actigraphy, wearable devices, polysomnography, or EEG-based measures, and should jointly report pain scores, opioid consumption, and sleep outcomes to better separate direct sleep effects from indirect analgesia-related benefits. Third, the perioperative administration of esketamine was conducted with varying protocols across studies (e.g., bolus/loading dose, maintenance infusion rate/duration and time of dosing during the perioperative period, co-administration with dexmedetomidine, use in patient-controlled intravenous analgesia) that may have contributed to variability in treatment effects. Fourth, few studies were available for many outcomes, particularly PSD on POD2 and POD7 and some adverse events, limiting statistical precision. Fifth, poorly reported allocation concealment in a subset of the included trials was a key reason for downgrading the certainty of evidence within GRADE. Sixth, the safety findings should be interpreted cautiously. Although no statistically significant increase in the adverse events analyzed in this review was observed, several safety outcomes were reported by only a small number of trials and involved few events. The analyses of dizziness and hypertension had wide confidence intervals, and dizziness showed moderate heterogeneity with very low certainty of evidence. In addition, broader perioperative esketamine meta-analyses have suggested increased risks of dizziness and hypertension. Therefore, the present review cannot exclude clinically meaningful hemodynamic or neuropsychiatric risks, and future trials should prespecify adverse-event definitions and systematically monitor blood pressure, dizziness, hallucinations, nightmares, dissociation, and other ketamine-related adverse effects. Finally, although trim-and-fill analysis did not significantly change the pooled estimate for POD1, Begg’s and Egger’s tests suggested possible publication bias or small-study effects, and thus the POD1 finding should be interpreted cautiously.

Clinically, the best evidence presented suggests adding perioperative esketamine can help in reducing sleep disturbance during early postoperative phases, especially on the first three postoperative days, without a significant increase in reported common adverse events. However, these results are not yet enough to recommend their routine adoption in clinical practice. Future trials should involve more diverse populations and multicenter settings, adopt more standardized and validated definitions of PSD, and better define the optimal dose, timing, and duration of perioperative esketamine. Future investigations should also assess longer-term sleep outcomes, functional recovery and neurocognitive endpoints to better define the contribution of esketamine to perioperative management of sleep.

## 5 Conclusion and recommendations

In summary, perioperative esketamine appears to reduce the incidence of PSD during the early postoperative period, most prominently from POD1 to POD3, while the absence of statistically significant increases in the adverse events examined in this meta-analysis should not be interpreted as definitive evidence of safety, because the available safety data were limited and broader perioperative esketamine reviews have suggested increased risks of dizziness and hypertension. However, this conclusion should be interpreted as evidence for a short-term reduction in binary PSD incidence rather than definitive evidence of comprehensive sleep improvement. The effects of esketamine on objective and specific sleep parameters, including total sleep time, sleep efficiency, wake after sleep onset, REM/NREM sleep architecture, and longer-term sleep recovery, remain insufficiently characterized. Because most incidence data were based on subjective sleep assessments, future studies should combine validated patient-reported scales with objective monitoring tools, such as actigraphy, wearable devices, polysomnography, or EEG-based measures, while also reporting pain scores and opioid consumption. Therefore, the available evidence does not justify the widespread perioperative use of esketamine in all surgical populations. Large, high-quality, multinational and multicenter trials are necessary to establish the reproducibility and generalizability of these effects across different healthcare systems, perioperative care pathways, and surgical populations. Future studies should standardize the definition and assessment of PSD, incorporate objective sleep monitoring such as actigraphy, polysomnography, or EEG-based measures, determine the optimal timing and dosing regimen of perioperative esketamine, and further explore its effects on functional recovery and postoperative neurocognitive outcomes.

## Data Availability

The original contributions presented in the study are included in the article/Supplementary Material, further inquiries can be directed to the corresponding authors.
