# Supplementary material for: Perioperative esketamine for prevention of postoperative sleep disturbance after anesthesia: a systematic review and meta-analysis of randomized controlled trials
Source: Front Pharmacol. 2026 Jun 3;17:1852647. doi: 10.3389/fphar.2026.1852647 (PMC13272463; doi:10.3389/fphar.2026.1852647)
Supplement: Supplementary file 1 [file Table1.docx]

**Supplementary Table 1. Detailed search strategies for all databases**

| **Database** | **Search Date** | **Language** | **Search Strategy** |
| --- | --- | --- | --- |
| PubMed | Up to March 20, 2026. | No restriction | ("esketamine"[Mesh] OR "S-ketamine"[tiab] OR "(S)-ketamine"[tiab] OR "esketamine"[tiab]) AND ("postoperative sleep disturbance"[tiab] OR "postoperative sleep"[tiab] OR "sleep quality"[tiab] OR "insomnia"[Mesh] OR "insomnia"[tiab]) AND ("anesthesia, general"[Mesh] OR "general anesthesia"[tiab]) |
| Embase | Up to March 20, 2026. | No restriction | ('esketamine'/exp OR 'S-ketamine':ti,ab OR '(S)-ketamine':ti,ab) AND ('postoperative sleep disturbance':ti,ab OR 'postoperative sleep':ti,ab OR 'sleep quality':ti,ab OR 'insomnia'/exp OR 'insomnia':ti,ab) AND ('general anesthesia'/exp OR 'general anesthesia':ti,ab) |
| Cochrane Library (CENTRAL) | Up to March 20, 2026. | No restriction | ("esketamine" OR "S-ketamine" OR "(S)-ketamine") AND ("postoperative sleep disturbance" OR "postoperative sleep" OR "sleep quality" OR "insomnia") AND ("general anesthesia") |
| CNKI | Up to March 20, 2026. | Chinese | 主题 = ("艾司氯胺酮" OR "S-氯胺酮" OR "(S)-氯胺酮") AND 主题 = ("术后睡眠障碍" OR "术后睡眠" OR "睡眠质量" OR "失眠") AND 主题 = ("全身麻醉" OR "全麻") |
