# Supplementary figures and images for: Perioperative esketamine for prevention of postoperative sleep disturbance after anesthesia: a systematic review and meta-analysis of randomized controlled trials
Source: Front Pharmacol. 2026 Jun 3;17:1852647. doi: 10.3389/fphar.2026.1852647 (PMC13272463; doi:10.3389/fphar.2026.1852647)

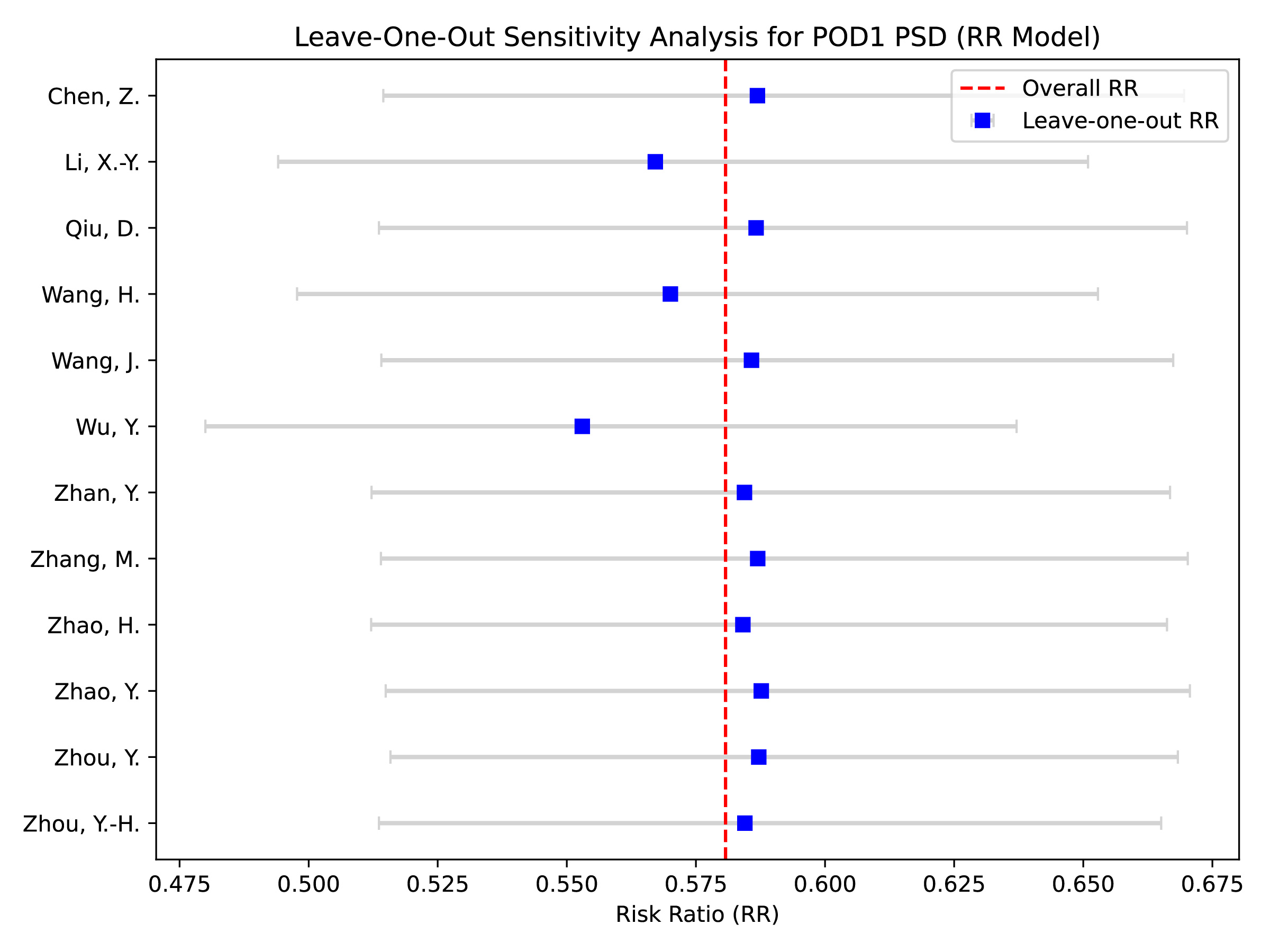

Supplement: Supplementary file 2 [file Image1.jpeg]

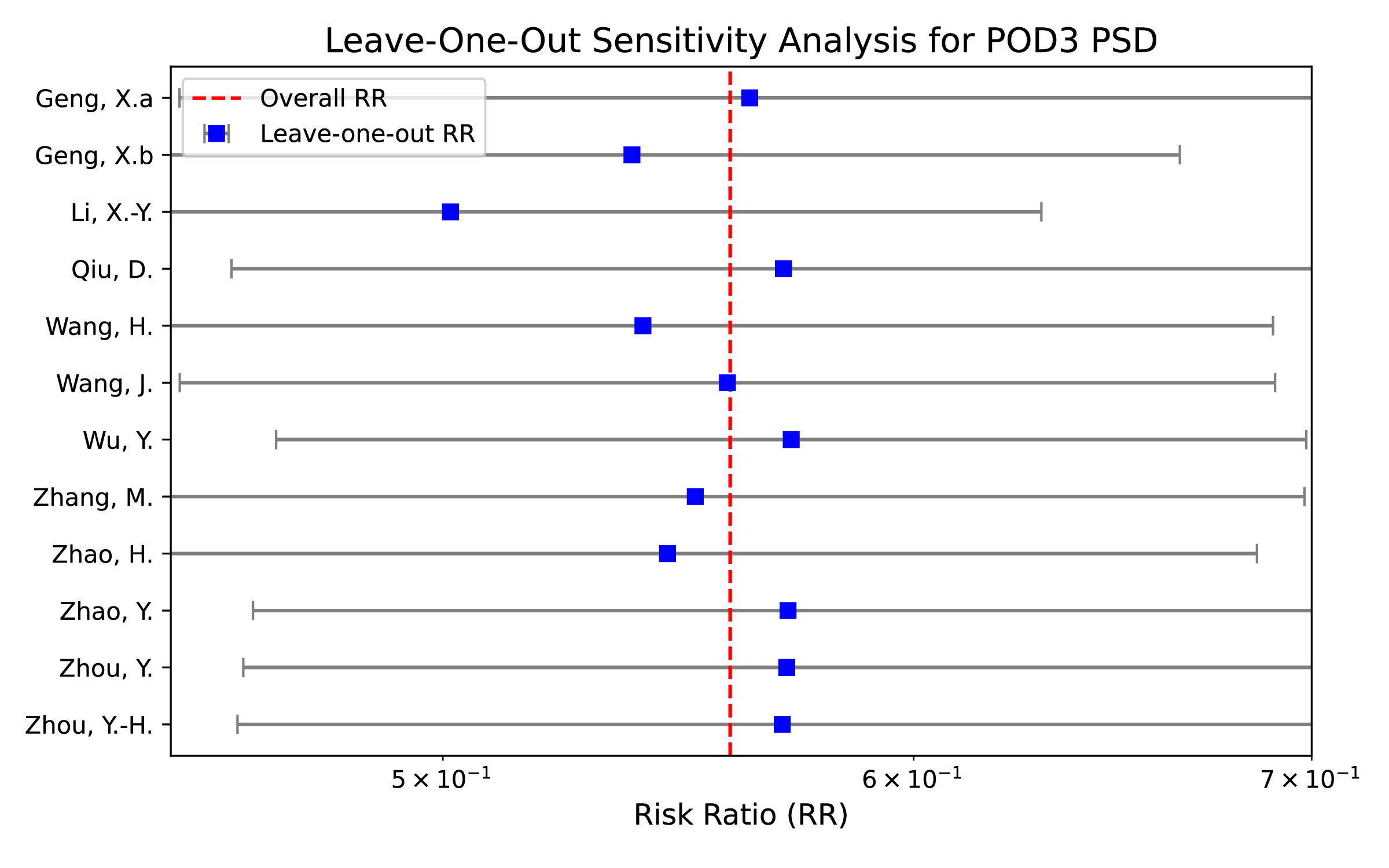

Supplement: Supplementary file 3 [file Image2.jpeg]
