# Supplementary material for: Perioperative esketamine for prevention of postoperative sleep disturbance after anesthesia: a systematic review and meta-analysis of randomized controlled trials
Source: Front Pharmacol. 2026 Jun 3;17:1852647. doi: 10.3389/fphar.2026.1852647 (PMC13272463; doi:10.3389/fphar.2026.1852647)
Supplement: Supplementary file 4 [file Table2.doc]

**Supplementary Table 1. Definitions of Postoperative Sleep Disturbance, Sleep Assessment Scales, and Esketamine Administration in Included Studies**

| **First Author** | **Sleep disorder outcome definition** | **Sleep Scale & Specific Score** | **Timing and administration method of esketamine** | **Included in RR meta-analysis for PSD incidence** |
| --- | --- | --- | --- | --- |
| Chen, H. | Postoperative AIS score used; explicit PSD cutoff not reported in current text | AIS: preop 4 (3, 6); POD1 4 (1, 6) | Intraoperative; continuous intravenous esketamine infusion as an anesthetic adjuvant during sedation anesthesia. | No. Continuous AIS score only; no explicit binary PSD incidence/cutoff available. |
| Chen, H. | Same as esketamine group | AIS: preop 4 (2, 7); POD1 5 (2, 9) | Intraoperative; matched saline infusion during sedation anesthesia. | No. Continuous AIS score only; no explicit binary PSD incidence/cutoff available. |
| Chen, Y. | Primary outcome = RCSQ total score on POD1; PSQI also assessed; no explicit binary PSD cutoff | RCSQ total: preop 64 (40–80), POD1 46 (32–68), POD2 70 (51.5–80); PSQI: POD30 NR | Intraoperative; IV loading dose followed by continuous IV infusion during surgery. | No. RCSQ/PSQI scores reported; no explicit binary PSD incidence/cutoff available. |
| Chen, Y. | Same as Group E | RCSQ total: preop 60 (41–79), POD1 54 (40–71), POD2 74 (60–80); PSQI: POD30 NR | Intraoperative; saline loading dose followed by continuous saline infusion during surgery. | No. RCSQ/PSQI scores reported; no explicit binary PSD incidence/cutoff available. |
| Chen, Z. | PSD = NRS-sleep ≥6 or AIS ≥6 | AIS and NRS-sleep on POD1 were lower than placebo; no significant difference by POD3 in current snippet | Both intraoperative and postoperative; IV dose at induction, low-dose intraoperative infusion, then added to PCIA for 48 h postoperatively. | Yes, POD1. Binary PSD event data based on NRS-sleep ≥6 or AIS ≥6 were available. |
| Chen, Z. | Same as esketamine group | AIS and NRS-sleep on POD1 higher than esketamine; POD3 difference not significant in current snippet | Both intraoperative and postoperative; saline placebo in the same schedule. | Yes, POD1. Binary PSD event data based on NRS-sleep ≥6 or AIS ≥6 were available. |
| Geng, X. | POSD = AIS >6 on at least one of POD1–3 | AIS: POD1 8 (4–9), POD3 5 (4–7) | Intraoperative; continuous IV infusion until placement of the drainage tube. | Yes, POD3. Binary POSD incidence was available. |
| Geng, X. | Same as above | AIS: POD1 9 (5–11), POD3 5 (4–5) | Intraoperative; no esketamine; dexmedetomidine infusion until placement of the drainage tube. | Yes, POD3. Binary POSD incidence was available. |
| Geng, X. | Same as above | AIS: POD1 8 (4.5–10), POD3 5 (4–8) | Intraoperative; combined continuous IV infusion until placement of the drainage tube. | Yes, POD3. Binary POSD incidence was available. |
| Geng, X. | Same as above | AIS: POD1 9 (5.5–10.5), POD3 8 (4.5–9.5) | Intraoperative; saline infusion until placement of the drainage tube. | Yes, POD3. Binary POSD incidence was available. |
| Li, D. | POSD incidence recorded; PSQI assessed preop day 1 and POD 1/2/5; PSG assessed preop day 1 and POD 1/2; explicit POSD cutoff not reported | PSQI:POD1 16. 23±1. 45 POD2 6. 23±1. 45 | Perioperative IV administration; single IV bolus followed by continuous IV infusion. Exact start time NR. | No. POSD incidence was mentioned, but extractable time-specific binary PSD event data for the meta-analysis were not available. |
| Li, D. | Same as observation group | PSQI:POD1 6. 87±1. 29 POD2 3. 71±1. 07 | Matched saline control; timing/method NR. | No. POSD incidence was mentioned, but extractable time-specific binary PSD event data for the meta-analysis were not available. |
| Li, X.-Y. | Preoperative sleep disorder: PSQI > 5. PSD: NRS ≥ 6 or AIS ≥ 6, assessed on POD 1/3/7 | PSQI: preop 12 (10–13.75); NRS sleep: POD1 5 (3–7), POD3 4 (2–6), POD7 4 (3–6); AIS: POD1 5 (3–8), POD3 4 (2–7), POD7 5 (3–7) | Intraoperative; continuous intravenous infusion during surgery. | Yes, POD1, POD3, and POD7. Binary PSD event data based on NRS ≥6 or AIS ≥6 were available. |
| Li, X.-Y. | Same as esketamine group | PSQI: preop 11 (10–15); NRS sleep: POD1 5 (4–7), POD3 4 (3–5), POD7 5 (3.5–6); AIS: POD1 6 (4–9), POD3 4 (3–6), POD7 6 (3–8.5) | Intraoperative; matched continuous saline infusion during surgery. | Yes, POD1, POD3, and POD7. Binary PSD event data based on NRS ≥6 or AIS ≥6 were available. |
| Ma, C.-B. | Sleep quality assessed by NRS 0–10; no explicit PSD/sleep-disorder cutoff reported | Sleep quality listed as a secondary outcome, but exact sleep-scale values were not captured in current snippet | Both intraoperative and postoperative; IV loading dose at induction, continuous intraoperative infusion, plus esketamine added to postoperative analgesia. | No. Sleep quality was assessed by NRS, but no explicit binary PSD incidence/cutoff was available. |
| Ma, C.-B. | Same as Group E | Sleep quality listed as a secondary outcome, but exact sleep-scale values were not captured in current snippet | Both intraoperative and postoperative; matched saline schedule. | No. Sleep quality was assessed by NRS, but no explicit binary PSD incidence/cutoff was available. |
| Pan, T. | Postoperative sleep disturbance incidence; PSQI subdomains (daytime dysfunction, sleep latency, sleep duration, sleep efficiency) on POD 1/3; explicit cutoff not reported | PSQI subscales, POD1: daytime dysfunction 1.23 ± 0.12, sleep latency 1.34 ± 0.08, sleep duration 1.43 ± 0.18, sleep efficiency 1.27 ± 0.12; POD3: 1.02 ± 0.19, 0.87 ± 0.02, 0.21 ± 0.12, 0.76 ± 0.19 | Intraoperative; continuous pump infusion until the end of surgery. | No. PSD incidence was reported, but the explicit cutoff and time-specific binary event definition for the meta-analysis were not clearly available. |
| Pan, T. | Same as A group | PSQI subscales, POD1: 1.87 ± 0.22, 1.79 ± 0.24, 1.95 ± 0.52, 1.54 ± 0.16; POD3: 1.52 ± 0.11, 1.55 ± 0.19, 1.28 ± 0.29, 1.21 ± 0.15 | Intraoperative; continuous pump infusion until the end of surgery. | No. PSD incidence was reported, but the explicit cutoff and time-specific binary event definition for the meta-analysis were not clearly available. |
| Pan, T. | Same as A group | PSQI subscales, POD1: 1.83 ± 0.28, 1.75 ± 0.19, 1.90 ± 0.11, 1.60 ± 0.19; POD3: 1.51 ± 0.11, 1.51 ± 0.12, 1.30 ± 0.11, 1.33 ± 0.18 | Intraoperative; continuous saline pump infusion until the end of surgery. | No. PSD incidence was reported, but the explicit cutoff and time-specific binary event definition for the meta-analysis were not clearly available. |
| Qiu, D. | PSD = NRS ≥6 or AIS ≥6 | NRS sleep: POD1 5 (4–5), POD3 4 (4–5); AIS: POD1 2 (2–3), POD3 2 (2–3) | Intraoperative; continuous IV infusion during surgery. | Yes, POD1 and POD3. Binary PSD event data based on NRS ≥6 or AIS ≥6 were available. |
| Qiu, D. | Same as esketamine group | NRS sleep: POD1 5 (5–6), POD3 4 (4–5); AIS: POD1 3 (2–6), POD3 3 (2–3) | Intraoperative; equivalent-volume saline infusion during surgery. | Yes, POD1 and POD3. Binary PSD event data based on NRS ≥6 or AIS ≥6 were available. |
| Wang, H. | Postoperative sleep disturbance = NRS ≥6 | Specific sleep scale score NR; postoperative sleep disturbance rate reported | Intravenous esketamine was given perioperatively; exact timing and whether bolus or continuous infusion were not clearly extractable. | Yes, POD1 and POD3. Binary PSD event data based on NRS ≥6 were available. |
| Wang, H. | Same as esketamine group | Specific sleep scale score NR; postoperative sleep disturbance rate reported | Intravenous saline control; exact timing/method not clearly extractable. | Yes, POD1 and POD3. Binary PSD event data based on NRS ≥6 were available. |
| Wang, J. | AIS assessed at preop day 1 and POD 1/3; PSD incidence assessed on POD 1/3; paper describes PSD as postoperative sleep-wake rhythm disturbance; explicit study cutoff for PSD incidence not clearly reported | AIS: preop 2 (1–4), POD1 4 (1.5–5), POD3 1 (0–2) | Intraoperative; continuous intravenous infusion during surgery. | Yes, POD1 and POD3. Binary PSD incidence was available. |
| Wang, J. | Same as esketamine group | AIS: preop 2 (1–4), POD1 5 (4–7), POD3 1 (0–3) | Intraoperative; matched continuous saline infusion during surgery. | Yes, POD1 and POD3. Binary PSD incidence was available. |
| Wang, P. | Sleep quality during the surgical night / first 24 h; explicit PSD cutoff not reported | Sleep quality score: preop 2 (1–4), surgical night 2 (1–2) | Intraoperative; single IV bolus followed by continuous IV infusion until wound closure. | No. Sleep quality score was reported, but no binary PSD incidence/cutoff was available. |
| Wang, P. | Same as esketamine group | Sleep quality score: preop 1 (1–2), surgical night 3 (1–4) | Intraoperative; saline bolus followed by continuous saline infusion. | No. Sleep quality score was reported, but no binary PSD incidence/cutoff was available. |
| Wei, Q. | PSQI measured before surgery, 0–24 h and 24–48 h after surgery; no explicit binary sleep-disorder cutoff | PSQI: preop 7 (4–9), 0–24 h 11 (6–13), 24–48 h 9 (5–12) | Postoperative; esketamine was administered through postoperative patient-controlled analgesia (PCA/PCIA). | No. PSQI scores were reported, but no explicit binary PSD incidence/cutoff was available. |
| Wei, Q. | Same as Es-D group | PSQI: preop 6 (4–9), 0–24 h 8 (4–11), 24–48 h 7 (3–10) | Postoperative; no esketamine; comparator analgesia was given through postoperative PCA/PCIA. | No. PSQI scores were reported, but no explicit binary PSD incidence/cutoff was available. |
| Wu, Y. | PSD = NRS ≥6 or AIS ≥6 | Preop PSQI 5 [4–6]; POD1 NRS-sleep 2 [2–9], AIS 8 [4–15]; POD2 NRS-sleep 1 [0–1], AIS 2 [0–3]; POD3 NRS-sleep 0 [0–0], AIS 0 [0–0] | Intraoperative; continuous intravenous infusion during surgery. | Yes, POD1, POD2, and POD3. Binary PSD event data based on NRS ≥6 or AIS ≥6 were available. |
| Wu, Y. | Same as esketamine group | Preop PSQI 5 [3–6]; POD1 NRS-sleep 9 [3–10], AIS 15 [10–19]; POD2 NRS-sleep 2 [1–3], AIS 6 [4–11]; POD3 NRS-sleep 1 [0–2], AIS 2 [2–6] | Intraoperative; matched continuous saline infusion during surgery. | Yes, POD1, POD2, and POD3. Binary PSD event data based on NRS ≥6 or AIS ≥6 were available. |
| Yuan, B. | Primary outcome = actigraphy-based sleep quality (TST, SE, WASO%) on preop night and POD1–2; no binary PSD cutoff | Actigraphy, PP, POD1: TST 475.5 ± 12.6 min; SE 67.1 ± 3.0%; WASO 27.2 ± 1.7% | Intraoperative; IV loading dose followed by continuous IV infusion during surgery. | No. Actigraphy-derived continuous sleep parameters were reported; no binary PSD incidence/cutoff was available. |
| Yuan, B. | Same as Group E1 | Actigraphy, PP, POD1: TST 480.7 ± 16.5 min; SE 68.0 ± 2.4%; WASO 26.9 ± 1.9% | Intraoperative; IV loading dose followed by continuous IV infusion during surgery. | No. Actigraphy-derived continuous sleep parameters were reported; no binary PSD incidence/cutoff was available. |
| Yuan, B. | Same as Group E1 | Actigraphy, PP, POD1: TST 469.3 ± 14.0 min; SE 65.8 ± 2.1%; WASO 28.9 ± 2.7% | Intraoperative; saline bolus followed by continuous saline infusion during surgery. | No. Actigraphy-derived continuous sleep parameters were reported; no binary PSD incidence/cutoff was available. |
| Zhan, Y. | POSD = PSQI ≥5 at 24 h postoperatively | PSQI: preop 3 (2–5), 24 h 2 (1–5), 72 h 1 (0–2.5) | Intraoperative; IV loading dose at induction followed by continuous IV maintenance infusion during anesthesia. | Yes, POD1. Binary POSD event data based on PSQI ≥5 at 24 h postoperatively were available. |
| Zhan, Y. | Same as esketamine group | PSQI: preop 4 (2–5), 24 h 4 (4–8), 72 h 3.5 (2.75–5) | Intraoperative; placebo administered in the same schedule as the esketamine group. | Yes, POD1. Binary POSD event data based on PSQI ≥5 at 24 h postoperatively were available. |
| Zhang, L. | RCSQ score on POD1 as primary outcome; no explicit binary PSD cutoff reported | RCSQ: POD1 43.5 ± 17.2, POD2 61.1 ± 11.6, POD3 63.8 ± 13.8 | Postoperative; esketamine was started in PCIA after fetal delivery and umbilical cord clamping. | No. RCSQ scores were reported, but no explicit binary PSD incidence/cutoff was available. |
| Zhang, L. | Same as Group E | RCSQ: POD1 52.6 ± 11.5, POD2 62.9 ± 13.8, POD3 66.1 ± 13.9 | Postoperative; esketamine was started in PCIA after fetal delivery and umbilical cord clamping. | No. RCSQ scores were reported, but no explicit binary PSD incidence/cutoff was available. |
| Zhang, L. | Same as Group E | RCSQ: POD1 31.9 ± 14.2, POD2 50.6 ± 16.2, POD3 58.7 ± 11.4 | Postoperative; PCIA without esketamine after fetal delivery and umbilical cord clamping. | No. RCSQ scores were reported, but no explicit binary PSD incidence/cutoff was available. |
| Zhang, M. | PSD = AIS ≥6 | AIS assessed on POD1 and POD3; continuous group score NR in main table | Intraoperative; continuous IV infusion throughout surgery. | Yes, POD1 and POD3. Binary PSD event data based on AIS ≥6 were available. |
| Zhang, M. | Same as esketamine group | AIS assessed on POD1 and POD3; continuous group score NR in main table | Intraoperative; matched saline infusion throughout surgery. | Yes, POD1 and POD3. Binary PSD event data based on AIS ≥6 were available. |
| Zhao, H. | AIS scores and PSD incidence reported on POD1/POD3; exact PSD cutoff not clearly shown in current extracted text | AIS: preop 4 (3–6), POD1 4 (3–5), POD3 3 (2–5) | Intraoperative; IV loading dose followed by continuous IV infusion during surgery. | Yes, POD1 and POD3. Binary PSD incidence was available. |
| Zhao, H. | Same as esketamine group | AIS: preop 4 (3–7), POD1 5 (4–9), POD3 4 (2–5) | Intraoperative; matched saline administration. | Yes, POD1 and POD3. Binary PSD incidence was available. |
| Zhao, Y. | PSD = sleep NRS ≥6 or AIS ≥6 | NRS sleep: preop 3 (2–3), POD1 3 (2–3.5), POD2 2 (2–3), POD3 2 (1–2), POD7 1 (0–1); AIS: preop 3 (2–3.5), POD1 4 (3–7), POD2 4 (3–5), POD3 3 (2–4), POD7 2 (1–3) | Postoperative; added to PCIA as a continuous background infusion for 48 h after surgery. | Yes, POD1, POD2, POD3, and POD7. Binary PSD event data based on sleep NRS ≥6 or AIS ≥6 were available. |
| Zhao, Y. | Same as esketamine group | NRS sleep: preop 3 (2–3), POD1 3 (3–4), POD2 3 (2–4), POD3 2 (1–3), POD7 1 (0–2); AIS: preop 3 (2–4), POD1 8 (4–9), POD2 4 (3–7), POD3 3 (2–5), POD7 2 (1–3) | Postoperative; PCIA without esketamine for 48 h after surgery. | Yes, POD1, POD2, POD3, and POD7. Binary PSD event data based on sleep NRS ≥6 or AIS ≥6 were available. |
| Zhou, Y. | Postoperative sleep dysfunction incidence and sleep time assessed at preop day 1 and POD 1/3; explicit postoperative sleep dysfunction cutoff not reported | Sleep time: POD1 6.8 ± 0.3 h, POD3 7.1 ± 0.4 h | Intraoperative; continuous intravenous infusion during surgery. | Yes, POD1 and POD3. Binary postoperative sleep dysfunction incidence was available. |
| Zhou, Y. | Same as esketamine group | Sleep time: POD1 5.7 ± 0.4 h, POD3 6.2 ± 0.3 h | Intraoperative; matched continuous intravenous saline infusion during surgery. | Yes, POD1 and POD3. Binary postoperative sleep dysfunction incidence was available. |
| Zhou, Y.-H. | Primary PSD definition: POD1 subjective sleep-quality NRS ≥6 | NRS sleep, POD1: 4 (2, 5) | Intraoperative; continuous IV infusion for 30 min immediately after anesthesia induction. | Yes, POD1 and POD3. Binary PSD incidence based on subjective sleep-quality NRS ≥6 was available. |
| Zhou, Y.-H. | Same as esketamine group | NRS sleep, POD1: 5 (3, 7) | Intraoperative; equivalent-volume saline infusion for 30 min immediately after anesthesia induction. | Yes, POD1 and POD3. Binary PSD incidence based on subjective sleep-quality NRS ≥6 was available. |

Note: “Included in RR meta-analysis for PSD incidence” indicates whether the study contributed binary event data to the pooled risk-ratio analysis of PSD incidence at the specified postoperative time point. Continuous sleep-scale scores, including AIS, PSQI, RCSQ, NRS-sleep scores, or actigraphy-derived parameters, were not transformed into dichotomous PSD events unless the original study explicitly reported binary PSD incidence according to its prespecified definition or cutoff.
